# PABPC1-induced stabilization of IFI27 mRNA promotes angiogenesis and malignant progression in esophageal squamous cell carcinoma through exosomal miRNA-21-5p

**DOI:** 10.1186/s13046-022-02339-9

**Published:** 2022-03-28

**Authors:** Ying Zhang, Chuangzhen Chen, Zhaoyong Liu, Huancheng Guo, Weiqing Lu, Wang Hu, Zhixiong Lin

**Affiliations:** 1 Department of Radiotherapy, Cancer Hospital of Shantou University Medical College, No. 7 Raoping Road, Shantou, Guangdong 515041 China; 2 Guangdong Provincial Key Laboratory for Breast Cancer Diagnosis and Treatment, Cancer Hospital of Shantou University Medical College, Shantou, Guangdong 515041 China; 3 Department of Orthopedics, First Affiliated Hospital of Shantou University Medical College, No.57 Changping Road, Shantou, 515041 Guangdong China

**Keywords:** ESCC, PABPC1, IFI27, eIF4G, miR-21-5p

## Abstract

**Background:**

Emerging evidence has demonstrated that RNA-binding protein dysregulation is involved in esophageal squamous cell carcinoma (ESCC) progression. However, the role of poly (A) binding protein cytoplasmic 1 (PABPC1) in ESCC is unclear. We therefore aimed to explore the functions and potential mechanisms of PABPC1 in ESCC progression.

**Methods:**

PABPC1 expression was characterized using immunohistochemistry and qRT-PCR in ESCC tissues and cell lines. Chromatin immunoprecipitation (ChIP) and luciferase reporter assays were used to detect histone acetylation in the promoter region of PABPC1. A series of *in vitro* and *in vivo* assays were further applied to elucidate the functions and underlying molecular mechanisms of PABPC1 in ESCC angiogenesis and malignant procession.

**Results:**

PABPC1 expression was upregulated in ESCC tissues compared with in normal esophageal epithelial tissues. Elevated PABPC1 expression was correlated with tumor cell differentiation and poor prognosis in patients. Sp1 and p300 cooperated to increase the level of H2K37ac in the PABPC1 promoter. Functionally, PABPC1 overexpression enhanced esophageal squamous cell proliferation and invasion by activating the IFN/IFI27 signaling pathway. PABPC1 interacted with eIF4G to increase the stability of IFI27 mRNA by competing with RNA exosomes in ESCC. Furthermore, PABPC1/IFI27 could increase miR-21-5p expression to enable exosomal delivery of miR-21-5p to human umbilical vein endothelial cells to increase angiogenesis via inhibiting CXCL10.

**Conclusion:**

PABPC1 plays a critical role in ESCC malignant progression by interacting with eIF4G to regulate IFI27 mRNA stability and promote angiogenesis via exosomal miR-21-5p/CXCL10. Taken together, our results suggest that PABPC1 is a promising therapeutic target for ESCC.

**Supplementary Information:**

The online version contains supplementary material available at 10.1186/s13046-022-02339-9.

## Background

Esophageal cancer is the sixth most common cause of cancer-related death worldwide and is therefore a major global health challenge. Esophageal squamous cell carcinoma (ESCC) is a highly prevalent cancer in China, which accounts for 90% of esophageal cancer globally [1]. Angiogenesis provides essential nutrients for tumor cell growth and metastasis, and has been shown to play a role in esophageal cancer [2]. Tumor cells can induce the formation of new blood vessels via cytokine, exosomes and other molecules [3, 4]. However, the tumorigenicity and angiogenesis of ESCC is not yet completely understood.

RNA-binding proteins (RBPs) have been identified as modulators of post-transcriptional mechanisms, including RNA splicing, transport, translation, and localization [5]. Increasing evidence suggests that RBPs have aberrant expression and function in multiple tumor types, such as glioma, hepatocellular carcinoma, and colorectal cancer [6]. Poly (A) binding protein cytoplasmic 1 (PABPC1), encoded within chromosome region 8q22.2–23, is a cytoplasmic-nuclear shuttling protein expressed in most eukaryotes. PABPC1 is an important RBP for the initiation of protein translation and mRNA decay. Biologically, PABPC1 is necessary for regulating vertebrate oocyte and early embryo translation as well as modulating the protein synthetic capacity of the mammalian heart [7, 8]. The role of PABPC1in cancer is not without controversy. Abnormal expression and function of PABPC1 have been detected in gliomas and hepatocellular carcinoma, where PABPC1 is highly expressed and enhances tumor cell proliferation [9, 10], although the combination of PABPC1 and BRCA1 has been suggested to have an anti-cancer function in breast cancer [11]. PABPC1 is correlated with clinical stage and survival of ESCC patients [12]. Despite the key role of PABPC1 in cancer progression, the mechanism by which it is regulated and participates in ESCC has not been clearly elucidated.

Interferon alpha (IFN-α) inducible protein 27 (IFI27), encoded within chromosome 14q32, has been reported to be involved in IFN-induced cell apoptosis, proliferation, and immune responses [13]. Furthermore, it has been shown to be an oncogene that is upregulated in various cancers, such as tongue squamous cell carcinoma, oral squamous cell carcinoma, and cholangiocarcinoma, and is correlated with poor survival [13–16]. Mechanically, IFI27 induces epithelial–mesenchymal transition (EMT) to promote cholangiocarcinoma metastasis [14]. IFI27 has been reported to be a putative cell proliferation marker and regulate cell cycle proteins, such as p21 and p53 [17]. Moreover, IFI27 mediated epithelial–mesenchymal transition (EMT) to promote cholangiocarcinoma metastasis [14]. IFI27 downregulation can inhibit tongue and oral squamous cell carcinoma cell proliferation and migration and invasion, and can promote cell apoptosis [16]. However, the function of IFI27 in ESCC and how PABPC1 mediates IFI27 have not been elucidated.

In this paper, we characterized PABPC1 expression and its clinical significance in ESCC. PABPC1 is highly expressed in ESCC and is correlated with a lower survival rate. PABPC1 promotes ESCC cell proliferation and invasion. Mechanistically, PABPC1 could increase IFI27 mRNA stability by interacting eIF4G to compete with the RNA exosome complex. PABPC1/IFI27 could increase miR-21-5p expression and promote exosomal miR-21-5p packaging to target human umbilical vein endothelial cells (HUVECs) and increase angiogenesis via inhibiting C-X-C motif chemokine 10 (CXCL10). Taken together, our results demonstrate that PABPC1 plays a tumor promoter role in ESCC and may therefore be a therapeutic target for treating ESCC.

## Materials and methods

### Patient samples and cell lines

A total of 190 ESCC tissue samples were obtained from patients who underwent cholecystectomy without prior radiotherapy or chemotherapy between 2015 and 2020 at Shantou University Medical College. Informed consent was obtained from all patients participating in the study. This study was approved by the Institutional Ethics Board of the Cancer Hospital of Shantou University Medical College (2021117). ESCC cell lines TE1, 81 T, KYSE410, KYSE180, KYSE450, KYSE150, KYSE510, and KYSE520 were cultured in high-glucose Dulbecco’s modified Eagle’s medium (DMEM, Gibco, Shanghai, China) or Roswell Park Memorial Institute (RPMI, Gibco) 1640 medium supplemented with 10% fetal bovine serum (FBS, Gibco). All cells were maintained in a humidified incubator (5% CO2) at 37°C. Immortalized esophageal epithelial NE1 cells were cultured in Eagle’s minimum essential medium (Gibco) supplemented with 10% FBS (Gibco). Cell lines were authenticated by short tandem repeat (STR) profiling and confirmed to be mycoplasma negative.

### Antibodies and western blotting

Cells were lysed in RIPA buffer (Beyotime, Jiangsu, China) containing a protease inhibitor cocktail (Sigma, St. Louis, CA, USA). Total protein was assessed using a BCA Protein Assay Kit (Beyotime). Western blot analysis was performed as previously described [18]. Antibodies against the following were used in this study, PABPC1 (1:1000, Abcam, SF, USA), IFI27, HA tag, Flag tag, TSG101, CD63, CD9, PCNA, Alix, Ki67, caspase 3, CXCL10, CD34, cleaved-PARP, PARP, eIF4G, cleaved caspase 9, caspase 9, ERK, p-ERK, IFI27, STAT3, p-STAT3, STAT1, p-STAT1, NF-kB, p-NF-kB, β-actin and GAPDH (all 1:1000, Cell Signaling Technology, MA, USA), EXOSC2 and EXOSC4 (both 1:1000, Santa Cruz, MA, USA).

### RNA extraction, RNA-sequencing, and quantitative real-time PCR (qRT-PCR)

Using an RNeasy Mini Kit (Qiagen, Valencia, CA, USA), total cellular RNA was extracted from cells. qRT-PCR was performed according to a previously published procedure using the SYBR Green system (Takara, Dalian, China) [19]. The primer sequences are shown in Table S1. For RNA-seq, the mRNA was amplified by PCR and sequenced using an Illumina NovaSeq 6000 (Gene Denovo Biotechnology Co., Guangzhou, China).

### Immunohistochemistry (IHC) and immunofluorescence (IF)

IHC staining was performed using the Envision Labeled Peroxidase System (Dako, Carpinteria, CA, USA) as described previously [19]. PABPC1 and IFI27 expression were analyzed based on the proportion and intensity of positively-stained tumor cells. The scores for intensity and fraction of positive cells were multiplied; scores of 0–4 were defined as low expression, and scores of 6–12 were defined as high expression. Immunofluorescence was performed as previously described [19]. Briefly, cells were first fixed in 3% paraformaldehyde, and then permeabilized with 0.5% Triton X-100. The cells were blocked with 5% FBS in PBS, followed by incubation with primary antibody, fluorescent secondary antibody (Invitrogen) and counterstained with DAPI (Beyotime) were then incubated with cells to visualize the targeted proteins and nuclei. The data were then analyzed by fluorescence microscopy.

### Drugs, plasmids, siRNA, and stable cell lines

Carbobenzoxy-Leu-Leu-leucinal (MG132), actinomycin D, and cycloheximide (CHX) were purchased from MCE (St. Louis, MO, USA). 5-Ethynyl-uridine (5-EU) and HDAC inhibitor sodium butyrate (NaBu) were purchased from Selleck (St. Louis, MO, USA). The siRNAs were designed and synthesized by GenePharma Company (Shanghai, China). Full-length PABPC1 plasmid, five truncated PABPC1 RRM1-RRM4 and MLLE plasmids, mutant PABPC1 ^M161A/D165K^ and RRM1 deletion PABPC1 plasmids, eIF4G, and IFI27 plasmids were purchased from Vigene (Shanghai, China). ESCC cell transfection was carried out using Lipofectamine 3000, Lipofectamine IMAX, and Opti-MEM (Invitrogen, Shanghai, China). For stable expression of PABPC1 or IFI27 in OS cell lines, lentivirus or short hairpin(sh) -RNA transfected cells were selected with puromycin (5 μg/mL, MCE) for 2 weeks.

### Co-immunoprecipitation (Co-IP)

Co-IP was performed using the relevant antibodies and protein A/G-conjugated Dynabeads (Beyotime) according to the manufacturer's instructions. In brief, cell lysates were incubated with antibodies overnight at 4 °C. Protein A/G-conjugated beads were added into the lysate at 4 °C for 2 h. Then, the beads were washed with lysis buffer or PBS and boiled in SDS loading buffer. Western blotting was used to detect the immunoprecipitated proteins.

### Cell proliferation, apoptosis, invasion, and migration assays

Cell proliferation was examined with a Cell Counting Kit-8 (CCK-8) according to the manufacturer’s instructions (Beyotime)**.** Apoptosis was determined by flow cytometry using Annexin V/PI staining according to the manufacturer’s protocol, and data analysis was performed using BD Accuri C6 software. Cell migration was evaluated by scratch wound healing assay where wounds were scratched on the monolayer of cells using a 200 μL pipette tip. After the cells had been cultured for 48 h, plates were washed once with fresh medium to remove non-adherent cells, and the plates were then photographed. Cell invasion and migration was tested using a transwell assay. Briefly, 100 μL Matrigel (Corning, CA, USA) was first added onto the bottom of the transwell chamber (Corning), and then 1 × 10^5^ cells in serum free medium were placed on the coated membrane in the top chamber and the bottom chamber was filled with DMEM with 10% FBS, then incubated for 24 h. The membrane was then fixed and stained with crystal violet. The cell imagines were taken and counted in 3 random fields with microscope.

### Human Umbilical Vein Endothelial Cells (HUVEC) tube formation assay

After co-cultured with different treatment of ESCC cells, 10^4^ HUVECs were cultured in μ-Slide Angiogenesis plate (ibidi, Germany) coated with 10 μL Matrigel (R&D Systems, Minneapolis, MN) for 6 h at 37 °C. The formation of capillary-like structures was captured under a light microscope. The degree of in vitro angiogenesis was represented the formed tube numbers scanned and quantitated in five low-power fields (100x).

### Dual reporter luciferase assay

Briefly, HEK293T or tumor cells (3 × 10^4^ cells per well) grown in a 24-well plate were co-transfected, with a luciferase reporter (200 ng per well), miR-21-5p mimic or inhibitor and 10 ng Renilla luciferase vector (pRL-CMV; Genomeditech, China), using Lipofectamine™ 3000 (Invitrogen). After 48 h, a dual reporter luciferase assay was performed according to the manufacturer’s instructions (Promega, Madison, MD, USA). The relative luciferase activity was expressed as the ratio of firefly luciferase activity to Renilla luciferase activity.

### RNA stability assay

To measure the stability of IFI27 mRNA, cells were treated with 5 μg/mL actinomycin D (MCE) to block transcription. Cells were collected at 0–24 h after addition of actinomycin D, and the total cellular RNA was isolated. qRT-PCR was performed to measure the half-life of RNA, and GAPDH mRNA was used as an internal control.

### Nascent RNA capture assay

To capture the newly synthesized RNA transcripts of IFI27 mRNA, a nascent RNA capture assay was carried out using the Click-IT Nascent RNA Capture Kit (Thermo Fisher Scientific, Waltham, MA, USA) according to the manufacturer’s protocol.

### Poly (A) tail-length assay

A poly (A) tail-length assay was performed using a Poly(A) Tail-Length Assay Kit (Invitrogen) according to the manufacturer’ s instructions. Briefly, total RNAs were first added to poly (G/I) and reverse transcribed. Then, the poly (A) length was detected by PCR using both gene-specific primers and the Universal PCR Reverse Primer (Table S1).

### Chromatin immunoprecipitation (ChIP) and RNA immunoprecipitation assay (RIP) assays

ChIP assays were performed using an EZ-ChIP kit (Millipore, Beijing, China). DNA was analyzed by qPCR with SYBR Green (Bio-Rad, Shanghai, China) on an ABI-7500 (Applied Biosystems, Shanghai, China) using the primers specified in Table S1. RIP assays were performed using an EZ-Magna RIP RNA-Binding Protein Immunoprecipitation kit (Millipore). Briefly, cells were collected and lysed in lysis buffer with protease inhibitors. The protein extract (500 μg) was incubated with 3 μg of PABPC1 antibody or IgG overnight at 4 °C. Approximately 30 μL of A/G protein magnetic beads were then added and the mixture was incubated at 4 °C for 4 h. After washing, co-immunoprecipitated RNAs were extracted. RNA fold enrichment is presented as percent input and compared with the IgG control.

### Biotin RNA pull-down assay

Biotin RNA pull-down experiments were performed using RNA pulldown kits (Bersin Bio, Guangzhou, China) according to the manufacturer’s instructions. Briefly, the cell lysates were incubated with 100 pmol of synthetic 5’-end biotin-modified IFI27 oligonucleotides overnight at 4 °C. After adding streptavidin agarose beads and incubating at 4 °C for 4 h, precipitates were washed five times and boiled in SDS buffer, followed by western blotting analysis.

### Exosome isolation and uptake assay

Exosome isolation was performed as previously described [18]. In brief, cancer cells were cultured with exosome-free FBS-containing media and grown to 70% confluence. Then, cells washed 3 times with PBS, and incubated for 24 h in serum-free media and the supernatant was collected. The supernatant was centrifuged at 3000 × g for 15 min to remove cells and cell debris and it was then mixed with ExoQuick exosome precipitation solution (SBI, Palo Alto, CA) and incubated overnight according to the manufacturer’s protocol. Then, the mixture was centrifuged at 1500 × g for 30 min at 4 °C. The pelleted exosomes were dissolved in PBS and were subsequently divided and transferred to RNase-free tubes to be stored or undergo electron microscopy, protein assays, RNA extraction, and use in *in vitro* or *in vivo* treatment. For exosome uptake experiments, exosomes were labeled with a PKH67 Green Fluorescent Cell Linker Kit (Sigma) following the manufacturer’s protocol. Then, 10 µg of exosomes was resuspended in 100 µl PBS and were added to 1 × 10^5^ HUVECs. HUVECs were harvested at 24 h for qRT-PCR and immunofluorescence analysis.

### Animal studies

For *in vivo* assays, each experimental group consisted of six 5-week-old male BALB/c-nu/nu mice. Briefly, 1.0 $\times$ 10^7^ cells stably overexpressing PABPC1 were suspended in 50 μL serum-free DMEM/Matrigel (1:1) and injected into the oxter of each mouse. The mice were euthanized at 5 weeks after injection, and the weight and size of the tumors were measured. For lung metastasis assays, 1.0 $\times$ 10^6^ PABPC1-overexpressing cells were suspended in 50 μL serum-free DMEM and injected into the tail veins of mice (*n* = 6/group). After 6 to 8 weeks, mice were euthanized, lung tissue was excised, and tumor nodules formed in the lung were counted and analyzed by H&E staining. The experimental protocol was approved by the Ethics Committee of Animal Experiments of the Cancer Hospital of Shantou University Medical College (SUMC2021-035).

### Statistical analysis

Statistical analyses were performed with SPSS version 22.0. Data are presented as the mean $\pm$ standard deviation (SD), and statistical significance was determined using unpaired Student’s *t*-tests. Survival curves were generated by the Kaplan–Meier method. *P*-values < 0.05 were considered to indicate statistical significance.

## Results

### PABPC1 is highly expressed in ESCC patient samples and correlates with poor prognosis

**Fig. 1:**
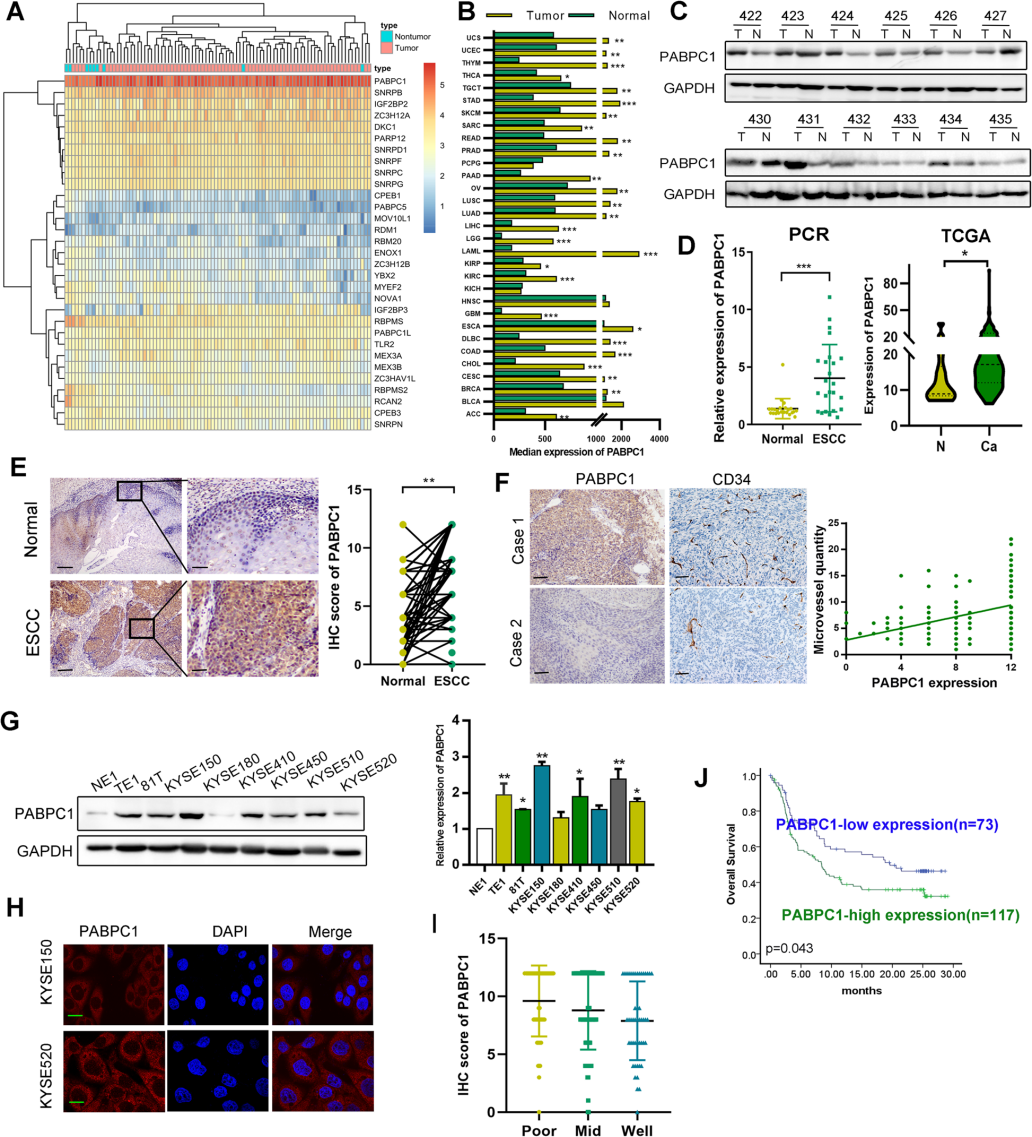
Expression of PABPC1 in ESCC tissues with its clinical significance. **A** Different expression levels of RNA-binding proteins, between normal and ESCC tissues, based on TCGA (https://cancergenome.nih.gov/). **B** Expression of PABPC1 in different types of cancer in the GEPIA database (http://gepia.cancer-pku.cn/). **C** Expression of PABPC1 in 24 pairs of ESCC samples (T) and corresponding non-tumor tissues (N) was detected by western blot analysis. **D** PABPC1 mRNA expression was detected by qRT-PCR in ESCC samples and non-tumor tissues (*n* = 48) and in TCGA. **E** Immunohistochemical staining of PABPC1 in primary ESCC samples and non-tumor tissues (*n* = 190) (scale bars: 200 µm, 50 µm). **F** Immunohistochemical staining of PABPC1 and CD34 in primary ESCC samples (*n* = 190) (scale bars: 200 µm). **G** Expression of PABPC1 in the immortalized esophageal cell line NE1 and ESCC cell lines (KYSE150, KYSE520, KYSE510, KYSE410, KYSE450, KYSE180, 81 T, and TE1) was detected by qRT-PCR and western blotting. **H** Expression of PABPC1 in ESCC cell lines KYSE150 and KYSE520 was detected by immunofluorescence (scale bars: 20 µm). **I** Expression of PABPC1 was higher in poorly differentiated than well differentiated tissues. **J** Comparison of overall survival of ESCC patients with high and low PABPC1 protein expression was determined by Kaplan–Meier survival analysis. Data represent the mean ± SD of 3 separate experiments. *p < 0.05, **p < 0.01, ***p < 0.001 by Student’s t test

Analyses of the expression levels of all RBPs in the esophageal cancer database from The Cancer Genome Atlas (TCGA) revealed higher PABPC1 expression in ESCC tissue than in normal esophageal epithelial tissue (Fig. 1A). We also examined PABPC1 expression levels in pan-cancers in the Gene Expression Profiling Interactive Analysis (GEPIA) database (http://gepia.cancer-pku.cn/index.html) and found that PABPC1 was expressed at higher levels in tumor tissue (Fig. 1B). In our ESCC cohort, PABPC1 expression was higher in ESCC tissue than in normal esophageal mucosa in the majority of cases (15/24), as determined by western blotting (Fig. 1C and S1A). Based on our data (*n* = 48) and TCGA, the RNA expression of PABPC1 was higher in ESCC compared with normal ESCC epithelia (Fig. 1D). As shown in Fig. 1E and F, the IHC staining score of PABPC1 was higher in ESCC tissue than in case-matched normal epithelial tissue (*n* = 190), and positively correlated with the microvessel density (MVD) detected by CD34 antibody. In addition, PABPC1 expression was higher in eight ESCC cell lines (KYSE150, KYSE520, KYSE510, KYSE410, KYSE450, KYSE180, 81 T, and TE1) compared with an immortalized normal esophageal cell line (NE1), as detected by western blotting and qRT-PCR (Fig. 1G). We assessed the subcellular localization of PABPC1 using immunofluorescence and observed detectable but weak nuclear PABPC1 and strong PABPC1 staining in the cytoplasm of KYSE150 and KYSE520 cells (Fig. 1H and S1B). According to the IHC scores, PABPC1 expression in ESCC tissue was positively and significantly correlated with tumor differentiation (Fig. 1I and Table 1). We also investigated the correlation between PABPC1 expression and survival time in ESCC patients, with a mean follow-up period of 12.5 months after surgery. Patients with high PABPC1 expression (*n* = 117) had a shorter median survival time (13.46 $\pm$ 1.12 months) after surgery than those with low PABPC1 expression (*n* = 73; 17.32 $\pm$ 1.38 months) (*p* = 0.043; log-rank test) (Fig. 1J). Similar results were found by analyzing ESCC data from TCGA (Figure S1C). Univariate and multivariate Cox regression analyses revealed that low PABPC1 expression was correlated with improved overall survival of ESCC patients (Figure S1D).

**Table 1:** Correlation between PABPC1 expression and clinical pathology parameters in ESCC

**Parameters**		**Case *n* = 190**	**Expression**		**Result**
			**Low (*n* = 73)**	**High (*n* = 117)**	
**Age**	≥ 57	87	36	51	
	< 57	103	37	66	*P* = 0.458
**Gender**	male	156	59	97	
	female	34	14	20	*p* = 0.703
**Differentiated**	poor	54	14	40	
	mid	88	43	45	
	well	48	16	32	*p* = 0.017*
**Tumor size**	> 3 cm	90	31	59	
	< 3 cm	100	42	58	*p* = 0.3
**Clinical stage**	II	24	11	13	
	III	100	39	61	
	IV	66	23	43	*p* = 0.629
**Adventitia invasion**	No	184	70	114	
	Yes	6	3	3	*p* = 0.677
**Lymph node metastasis**	No	102	37	65	
	Yes	88	36	52	*p* = 0.551
**Distal metastasis**	No	178	70	108	
	Yes	12	3	9	*p* = 0.377

### Sp1/p300 regulates PABPC1 promoter acetylation in ESCC

**Fig. 2:**
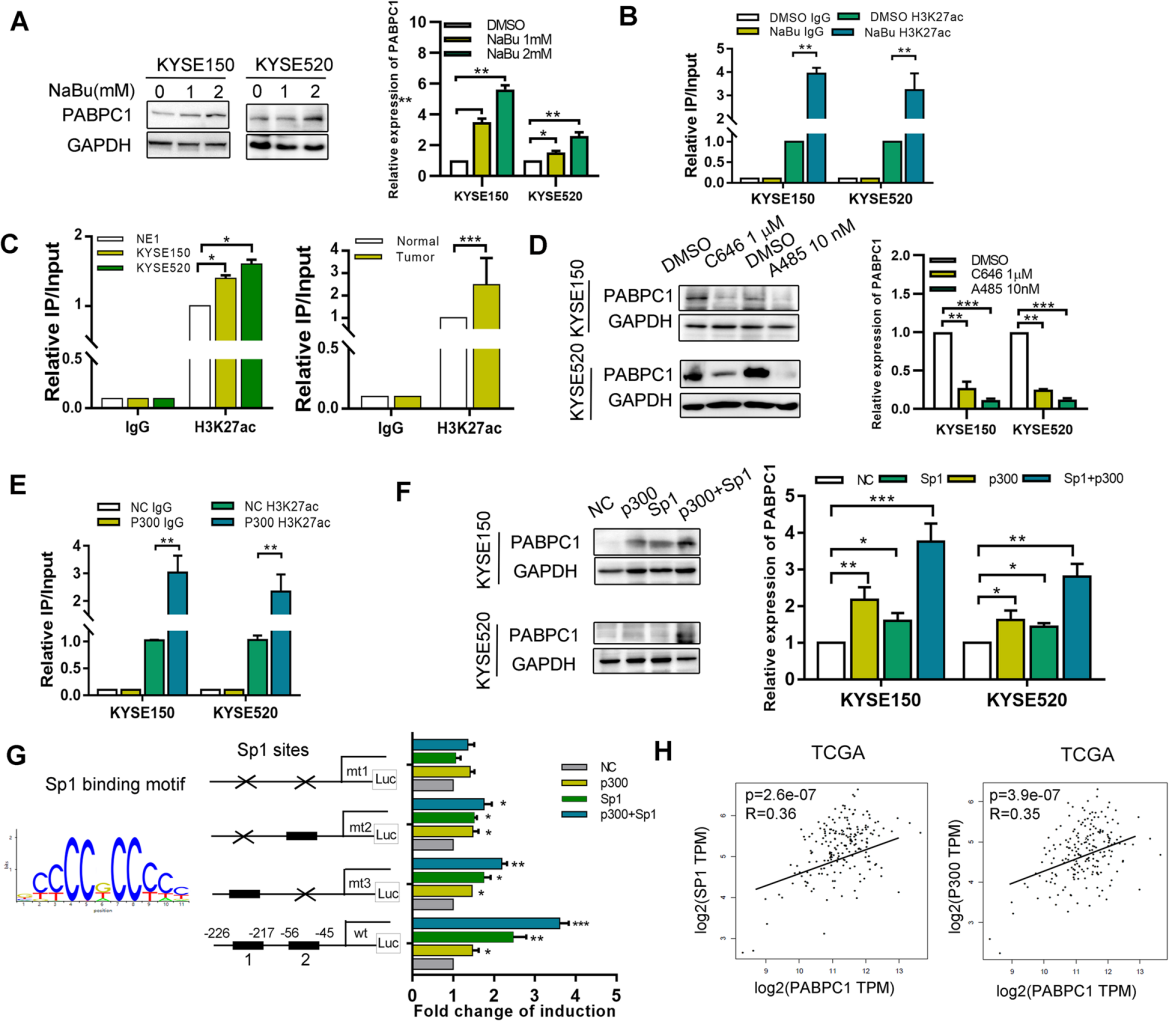
P300/Sp1 upregulates PABPC1 expression by modulating PABPC1 promoter H3K27 acetylation. **A** Expression of PABPC1 was detected by western blotting and qRT-PCR following treatment of KYSE150 and KYSE520 cells with different doses of NaBu. **B** ChIP assay demonstrating that H3K27 acetylation occurred in the PABPC1 promoter in ESCC cell lines following treatment with NaBu (2 mM). **C** ChIP assay demonstrating that H3K27 acetylation occurred in the PABPC1 promoter in the immortalized esophageal cell line NE1 and KYSE150 and KYSE520 ESCC cell lines**.** ChIP assay demonstrating that H3K27 acetylation occurred in the PABPC1 promoter in normal and ESCC tissues (*n* = 3). **D** Expression of PABPC1 was detected by western blotting and qRT-PCR following treatment of KYSE150 and KYSE520 cells with C646 or A485. **E** ChIP assay demonstrating that H3K27 acetylation occurred in the promoter of PABPC1 in ESCC cell lines following transfection of p300. **F** Expression of PABPC1 was detected after co-transfection of Sp1 and p300 plasmids and detection by western blot and qRT-PCR. **G** HEK293T cells were co-transfected with different combinations of wild-type (wt) and Sp1-site-mutated reporter constructs (mt 1, mt 2, and mt 3), p300 and control. The relative luciferase activity was analyzed. **H** Correlation of PABPC1 and Sp1 or p300 in TCGA was analyzed. Data represent the mean ± SD of 3 separate experiments. *p < 0.05, **p < 0.01, ***p < 0.001 by Student’s t test

We explored the probable mechanisms of high PABPC1 expression in ESCC by analyzing data from the Encyclopedia of DNA Elements (ENCODE) database (http://genome.ucsc.edu/) (Figure S2A). To determine whether histone acetylation is involved in human PABPC1 transcriptional regulation, we treated KYSE150 and KYSE520 cells with the histone deacetylase (HDAC) inhibitor NaBu. PABPC1 mRNA levels and H3K27ac enrichment in the promoter region of PABPC1 were remarkably increased in NaBu-treated ESCC cells compared with that in untreated controls (Fig. 2A, B). As shown in Fig. 2C, higher H3K27Ac levels were observed in the PABPC1 promoter region in ESCC tissues (*n* = 3) and cells (KYSE150 and KYSE520) than in normal tissues and normal NE1 cells. These results suggest that histone acetylation might regulate PABPC1 expression in transcriptional regulation.

Therefore, we hypothesized that certain histone acetylation-modifying enzymes or co-activators might be involved in PABPC1 regulation. As shown in Figs. 2D and S2B, two histone acetyltransferase p300 inhibitors (C646 and A485) decreased, while p300 transfection increased the expression of PABPC1 in a dose-dependent manner. p300 overexpression also increased the levels of H3K27ac in the promoter region of the PABPC1 gene, as identified by chromatin immunoprecipitation (ChIP) (Fig. 2E). These results indicate that p300 might serve as an epigenetic regulator of PABPC1 transcription. We further investigated the transcription factor that combined with p300 to regulate H3K27 acetylation at the PABPC1 promoter. There are two putative Sp1 binding sites close to each other within the PABPC1 promoter region (-226/-217 and -56/-45 bp). Western blot analysis showed that Sp1 increased PABPC1 expression in a dose-dependent manner (Figure S2C). As expected, western blotting revealed strong PABPC1 expression in the p300 and Sp1 co-transfected cells, which was substantially stronger than the expression in cells transfected with the p300 or Sp1 plasmids alone (Fig. 2F). To evaluate whether the two Sp1-binding sites in the PABPC1 promoter region contribute to Sp1-mediated p300 activity, we constructed three reporter plasmids with mutations in the -226/-217 Sp1 site, the -56/-45-Sp1 site, and in both sites. Relative to wildtype promoter, all mutations reduced the transcription activity of p300 and Sp1 on the PABPC1 promoter, and the double-mutated plasmid was associated with substantially decreased activity of p300 and Sp1 (Fig. 2G). Furthermore, TCGA database analysis revealed a positive correlation between p300 and PABPC1 and between Sp1 and PABPC1 expression in ESCC tissues (Fig. 2H). Taken together, these results indicate that the binding of Sp1 with p300 leads to H3K27ac enrichment at the PABPC1 promoter to result in activation of PABPC1 transcription.

### PABPC1 promotes ESCC cell proliferation, migration and angiogenesis *in vivo* and *in vitro*

**Fig. 3:**
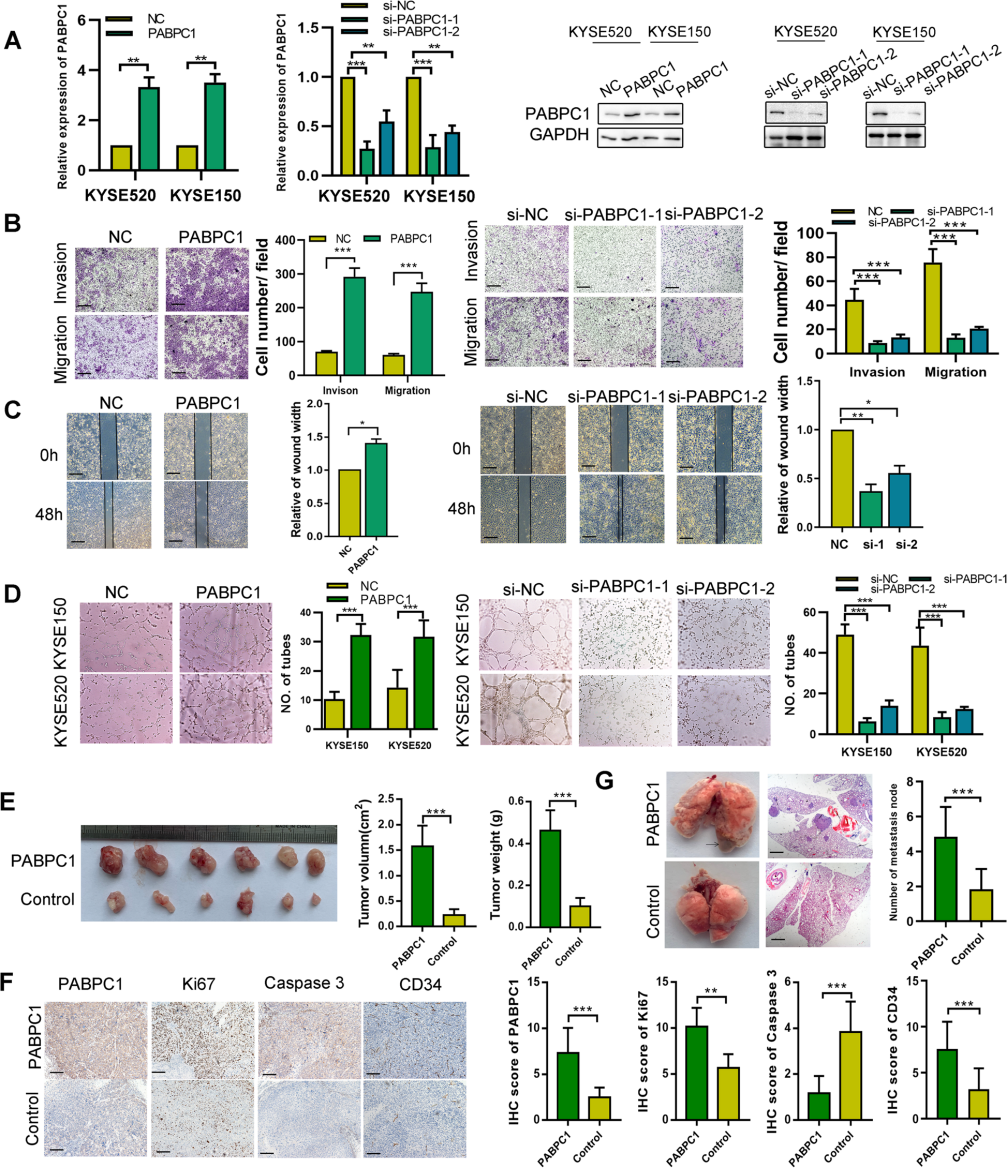
PABPC1 promotes cell proliferation, migration, and invasion *in vivo* and *in vitro*. **A** Confirmation of PABPC1 plasmid and siRNA transfection by western blot and qRT-PCR. **B** Cell invasion and migration was detected by transwell assay in PABPC1-overexpressing or knockdown ESCC cell lines (scale bar: 200 µm). **C** Cell migration was detected by wound healing assay in PABPC1-overexpressing or knockdown ESCC cell lines (scale bar: 200 µm). **D** Capillary tube formation assay of HUVECs co-cultured with PABPC1-overexpressing or knockdown ESCC cells. **E** Effects of PABPC1 overexpression on ESCC progression in subcutaneous xenograft mouse models. Tumor growth volume and weight of xenografts derived from control or PABPC1 cells are shown. **F** Representative photographs of immunohistochemical staining of tumor tissues from mice inoculated with PABPC1-overexpressing and control cells (scale bar: 200 µm). **G** Representative images of mass and H&E-stained sections from metastatic nodules in the lung (scale bar: 200 µm). Data represent the mean ± SD of 3 separate determinations. *p < 0.05, **p < 0.01, ***p < 0.001 by Student’s t test

We then investigated the biological effects of PABPC1 in ESCC. The efficiency of PABPC1 plasmid and siRNA transfection was confirmed by qRT-PCR and western blot (Fig. 3A). By using CCK-8 assays, we observed that the growth rate of KYSE520 and KYSE150 cells overexpressing PABPC1 was increased compared to that of the negative control, while the growth rates of PABPC1 knockdown cell lines was decreased (Figure S3A). Furthermore, colony numbers were increased in PABPC1-overexpressing cell lines, but decreased in knockdown cell lines (Figure S3B). To further explore the effect of PABPC1 on apoptosis, western blot and flow cytometry were performed in ESCC cells. The results showed a significant decrease in cleaved PARP and caspase 9 expression, and in the percentage of apoptotic cells when cells overexpressed PABPC1 (Figure S3C, D). Next, transwell and wound healing assays results showed that PABPC1 overexpression promoted ESCC cell invasion and migration, whereas PABPC1 knockdown inhibited these functions (Fig. 3B, C). We further investigated whether PABPC1 could promote tumor angiogenesis. We observed that PABPC1-overexpressing ESCC cells causeda substantial increase in the tube formation of HUVECs when compared with control cells, while knockdown PABPC1 decreased the tube formation (Fig. 3D). Nude mice were subcutaneously injected with KYSE150 cells stably overexpressing PABPC1, and tumor growth was larger compared with control cells. As shown in Fig. 3E, tumors derived from the PABPC1 groups were larger and weighed more than those in the KYSE150 control groups. Moreover, the KYSE150-PABPC1 groups had higher expression of Ki-67 and CD34, and decreased levels of cleaved caspase-3 compared with the control group (Fig. 3F). *In vivo* studies showed that ectopic overexpression of PABPC1 largely inhibited ESCC cell migration (Fig. 3G). Taken together; these data suggest that PABPC1 increases ESCC cell proliferation, invasion, and migration *in vitro*, and tumor formation *in vivo*.

### IFI27 is a key factor in PABPC1-mediated ESCC tumorigenesis

**Fig. 4:**
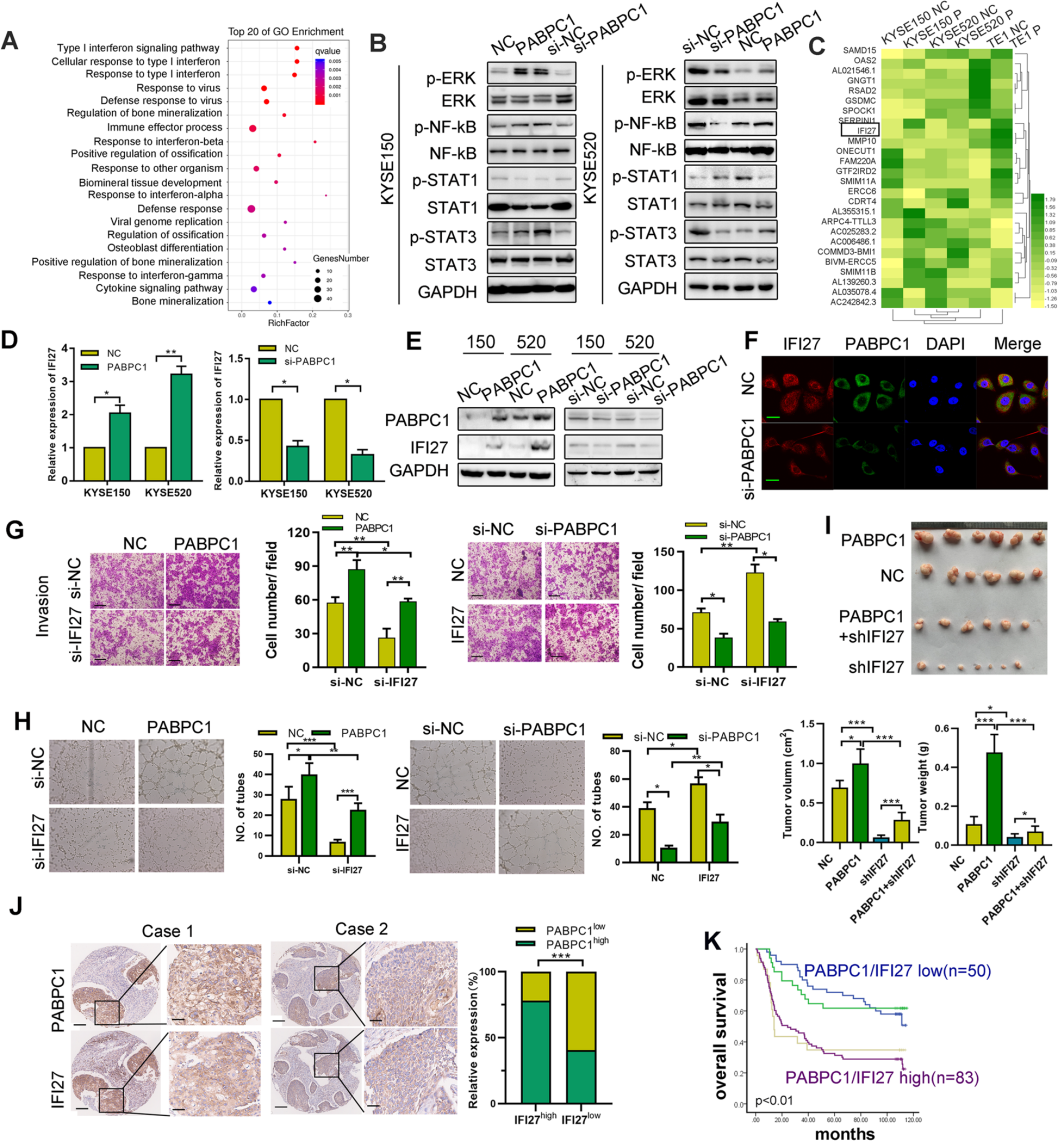
PABPC1 mediates IFI27 to promote ESCC malignancy. **A** Scatter plot of the top 20 KEGG pathway enrichment of DEGs after PABPC1 transfection. The enrichment factor is the ratio of the DEG number to the background number in a corresponding pathway. The size of the dots represents the number of genes, and the color of the dots represents the range of the q-value. **B** Expression of the indicated protein was detected in PABPC1-overexpressing or knockdown ESCC cell lines. **C** The top DEGs are listed in the heatmaps. **D** IFI27 mRNA expression levels were determined by qRT-PCR in ESCC cells overexpressing PABPC1 or with PABPC1 knockdown. **E** IFI27 protein expression levels were determined by western blotting in ESCC cells overexpressing PABPC1 or with PABPC1 knockdown. **F** IFI27 and PABPC1 protein expression was detected by immunofluorescence (scale bar: 20 µm). **G** Transwell assays were performed to determine cell invasion by the indicated transfected KYSE150 cells (scale bars: 200 µm). **H** Capillary tube formation assay of HUVECs treated with exosomes from PABPC1-overexpressing or PABPC1 knockdown ESCC cells. **I** Tumor volume and weight of xenografts derived from the indicated KYSE150 cell group (one of the mice in the shIFI27 group had two masses). **J** Expression of PABPC1 and IFI27 was detected by immunohistochemistry in a cohort of ESCC patients (scale bars: 200 µm, 100 µm). Expression of PABPC1 was positively correlated with IFI27. **K** Kaplan–Meier analysis indicating the patients with both high expression of PABPC1 and IFI27 have the worst survival, and patients with weak expression of both PABPC1 and IFI27 have better survival. Data represent the mean ± SD of 3 separate determinations. *p < 0.05, **p < 0.01, ***p < 0.001 by Student’s t test

We performed RNA-sequencing (RNA-seq) to explore the downstream genes potentially involved in PABPC1-induced ESCC proliferation and invasion. We identified 440 differentially expressed genes (DEGs) that were upregulated and 359 that were downregulated between the control and PABPC1 overexpressing groups. Selected DEGs from the RNA-seq results were confirmed by qRT-PCR (Figure S4A). Analysis of DEGs via Gene Ontology (GO) and the Kyoto Encyclopedia of Genes and Genomes (KEGG) indicated that PABPC1 regulates the interferon (IFN) signaling pathway (Fig. 4A). We detected the expression of key mediators of IFN pathways, and found that PABPC1 increased STAT3, ERK, and NF-kB phosphorylation, while it decreased STAT1 phosphorylation (Fig. 4B). Upregulation of IFI27, a key regulator in the IFN-α signaling pathway, was among the most significantly altered DEGs upregulated by PABPC1 (Fig. 4C). The mRNA and protein expression level of IFI27 was also regulated by PABPC1, as detected by qRT-PCR, western blotting, and immunofluorescence (Fig. 4D-F). Then, we sought to determine whether PABPC1 promotes ESCC tumorigenesis via IFI27. We knocked down IFI27 in PABPC1-overexpressing cells, or knocked down PABPC1 in IFI27-overexpressing KYSE150 cells, and detected cell apoptosis, proliferation, invasion, migration and angiogenesis (Figs. 4G, H and S4B-F). IFI27 knockdown dampened PABPC1-mediated increases in STAT3, ERK, and NF-kB phosphorylation (Figure S4G). To further evaluate the effects of PABPC1 and IFI27 on tumor growth, we established xenograft mouse models of ESCC (6 mice per group). The size and weight of xenograft tumors expressing PABPC1 were significantly larger than those of the negative control (NC), and were further reduced when PABPC1 expression was combined with shIFI27 transfection (Fig. 4I). Furthermore, the most significant reduction in tumor size and weight was observed with shIFI27 alone. Tumors with high PABPC1 expression had markedly increased Ki-67 levels but low caspase 3 (Figure S4H). The increase in Ki-67 was attenuated in the xenografts treated with shIFI27. Taken together, these data suggest that PABPC1-induced tumor progression is associated with IFI27 expression.

To evaluate the clinical relevance of our findings *in vivo* and *in vitro*, we explored IFI27 expression in public databases and our ESCC patient tissue cohort. IFI27 expression was higher in cancer tissues than in normal tissues in most cancer types in GEPIA database (Figure S5A). IFI27 expression was significantly higher in ESCC tissues compared to paired non-tumor tissues in the GSE20347 (*n* = 34) and GSE23400 cohorts (*n* = 106), as well as in our ESCC cohort detected by western blotting (*n* = 24) and immunohistochemistry (*n* = 190) (Figure S5B-D). Survival analysis revealed that patients in the high IFI27 group had shorter overall survival than those in the low IFI27 group in both our cohort and TCGA (Figure S5E). We then used IHC staining of 190 ESCC tissues to explore whether a clinical correlation exists between PABPC1 and IFI27 expression. We found that the IFI27 immunohistochemistry score was positively correlated with PABPC1, with approximately 78.3% of tumor tissues (83/106) with high IFI27 expression showing high PABPC1 staining, and 59.5% (50/84) of those with low IFI27 expression displaying low PABPC1 staining (Fig. 4J). Furthermore, survival analysis illustrated that high expression of both PABPC1/IFI27 predicted the poorest overall survival, while patients with low PABPC1/IFI27 expression had best overall survival (*p* < 0.01) (Fig. 4K).

### PABPC1 interacts with eIF4G to protect IFI27 mRNA from degradation

**Fig. 5:**
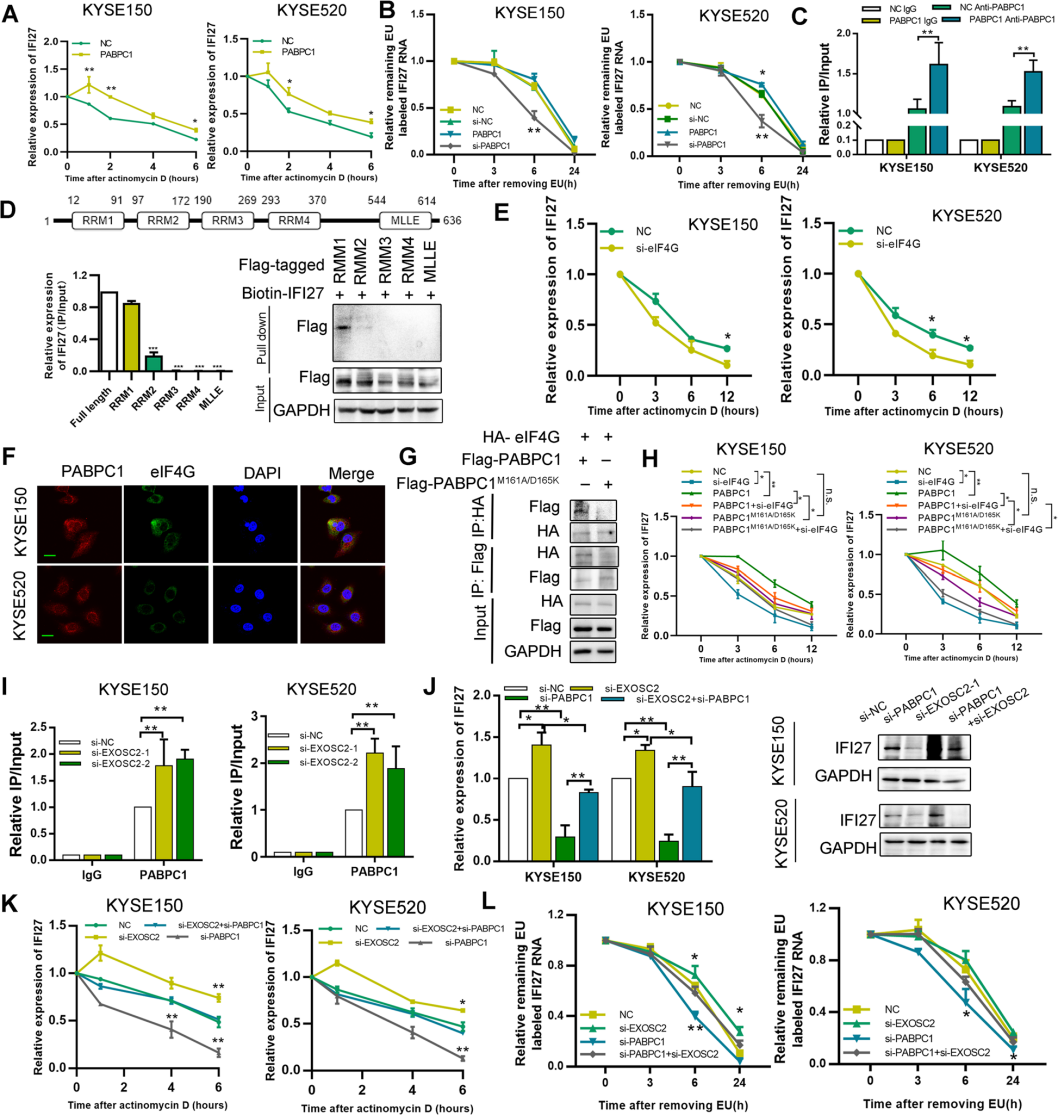
PABPC1 interacts with eIF4G to prevent IFI27 mRNA degradation. **A** PABPC1-overexpressing and control cells were treated with the transcription inhibitor actinomycin D (5 mg/mL). IFI27 mRNA levels were quantified using qRT-PCR. **B** 5-Ethynyluridine (EU) labeling assay to determine IFI27 mRNA stability following PABPC1 overexpression or knockdown. **C** RNA immunoprecipitation (RIP) assay, using PABPC1 antibody, demonstrating enrichment of IFI27 mRNA compared to the negative control IgG (upper panel). RNA pull-down was performed using biotin-labeled IFI27 RNA (lower panel). **D** Flag-tagged RRM1-4 and MLLE were transfected into HEK293T cells, then RIP and RNA pull-down were performed to detect the binding of IFI27 mRNA and PABPC1, respectively. **E** IFI27 mRNA expression levels were determined in ESCC cells following si-eIF4G transfection after actinomycin D (5 mg/mL) treatment. **F** The co-localization of PABPC1 and eIF4G was detected by immunofluorescence (Scale bar: 20 µm). **G** Co-binding of flag-tagged wildtype PABPC1, PABPC1^M161A/D165K^ and HA-tagged eIF4G was detected by co-immunoprecipitation in HEK293T cell. **H** Expression of IFI27 mRNA in KYSE150 and KYSE520 cells was detected after the indicated transfection and treatment with actinomycin D (5 mg/mL). **I** QRT-PCR analysis demonstrating IFI27 mRNA levels following knockdown of EXOSC2 cells with two separate siRNAs in KYSE150 and KYSE520 cells. **J** QRT-PCR and western blotting were performed to detect the expression of IFI27 in the indicated transfected ESCC cells. **K** ESCC cells transfected with the indicated siRNAs were treated with actinomycin D (5 mg/mL), and IFI27 mRNA stability was assessed. **L** 5-EU labeling assay to determine IFI27 mRNA stability under PABPC1 or EXOSC2 knockdown conditions. Data represent the mean ± SD of 3 separate determinations. *p < 0.05, **p < 0.01, ***p < 0.001 by Student’s t test

We next sought to gain mechanistic insight into how PABPC1 affects IFI27 expression. First, PABPC1 did not affect IFI27 protein stability, gene transcription activity, poly (A) tail length, or mRNA synthesis, as detected by luciferase reporter gene assay, poly(A) tail length, and nascent RNA capture assays (Figure S6A-D). Since PABPC1 mediated mRNA stability, we hypothesized that PABPC1 increases IFI27 mRNA expression by increasing its stability. We treated ESCC cells with the RNA synthesis inhibitor actinomycin D (5 μg/mL) and found that the half-lives of IFI27 mRNA were significantly shorter in PABPC1-depleted cells compared to in control cells. Meanwhile, PABPC1 expression could increase IFI27 mRNA stability compared to in control cells (Fig. 5A and S6E). To exclude the possibility that actinomycin D itself could be affecting IFI27 mRNA stability, we also measured RNA stability using the 5-ethynyluridine (EU) labeling (0.1 mM) and release method. We consistently observed a significant decrease in 5-EU-labeled IFI27 mRNA after PABPC knockdown (Fig. 5B). These results indicate that PABPC1 regulates IFI27 expression at the posttranscriptional level.

We confirmed the binding of IFI27 mRNA and PABPC1 by biotin-RNA pull-down assays (Figures S6F). IFI27 mRNA enrichment was significantly increased in the PABPC1 group compared to the control group by RIP assay (Figs. 5C). To detect the possible RNA binding domain via which PABPC1 interacts with IFI27 mRNA, we established five Flag-tagged plasmids containing N-terminal RNA recognition motifs 1–4 (RRMs) and the C-terminal MLLE of PABPC1. As shown in Fig. 5D, the RRM1 domain had strong binding affinity for IFI27 mRNA, whereas the RRM2 domain had weak binding affinity for IFI27 mRNA. PABPC1 interacts with eukaryotic initiation factor 4G (eIF4G) to trigger mRNA circularization and protect mRNA from degradation [20]. To determine whether eIF4G is involved in PABPC1-mediated IFI27 mRNA degradation, we induced eIF4G knockdown and found that the half-life of IFI27 RNA was significantly reduced compared to control (Fig. 5E). Then, we confirmed the colocalization of eIF4G and PABPC1 by immunofluorescence (Fig. 5F). The binding between eIF4G and PABPC1, as well as between IFI27 mRNA and PABPC1, was weaker in response to eIF4G knockdown (Figure S6G-K). To confirm the function of the PABPC1-eIF4G-RNA ternary complex in mRNA decay, we introduced two-point mutations into PABPC1 (PABPC1^M161A/D165K^) that abolish binding to eIF4G (Fig. 5G). Notably, PABPC1^M161A/D165K^ transfection no longer suppressed mRNA degradation, and IFI27 mRNA levels remained unchanged in response to eIF4G knockdown (Fig. 5H). To further confirm our findings, we constructed a PABPC1 plasmid with an RRM1 domain deletion, ΔRRM1 PABPC1. As shown in Figure S6L and M, ΔRRM1 PABPC1 abolished PABPC1 binding to eIF4G, and did not affect the mRNA expression and half-life of IFI27. Interestingly, we found that IFI27 knockdown decreased the binding of PABPC1 and eIF4G (Figure S6M). These results indicate that the interaction between PABPC1 and eIF4G is critical for protecting IFI27 mRNA from degradation.

EXOSC2 (exosome component 2), an important catalytic component of the RNA exosome complex, plays a vital role in regulating RNA degradation [21]. As expected, the decreased stability of IFI27 mRNA could also be rescued by EXOSC2 knockdown (Figure S7A-B). Using a RIP assay, we confirmed that EXOSC2 interacts with IFI27 RNA, and EXOSC2 knockdown increased the binding of PABPC1 with IFI27 RNA (Fig. 5I). This is similar to the manner in which PABPC1 knockdown increased the binding affinity of EXOSC2 with IFI27 mRNA (Figures S7C). In addition, EXOSC2 knockdown reversed the decrease in IFI27 expression levels and RNA half-life caused by PABPC1 knockdown (Fig. 5J-L). Similar results were found for EXOSC4, a core component of the RNA exosome (Figure S7D-F). These findings demonstrate that PABPC1 competed with the RNA exosome to prevent IFI27 mRNA degradation.

### PABPC1/IFI27 regulate angiogenesis through exosome-mediated transfer of miR-21

**Fig. 6:**
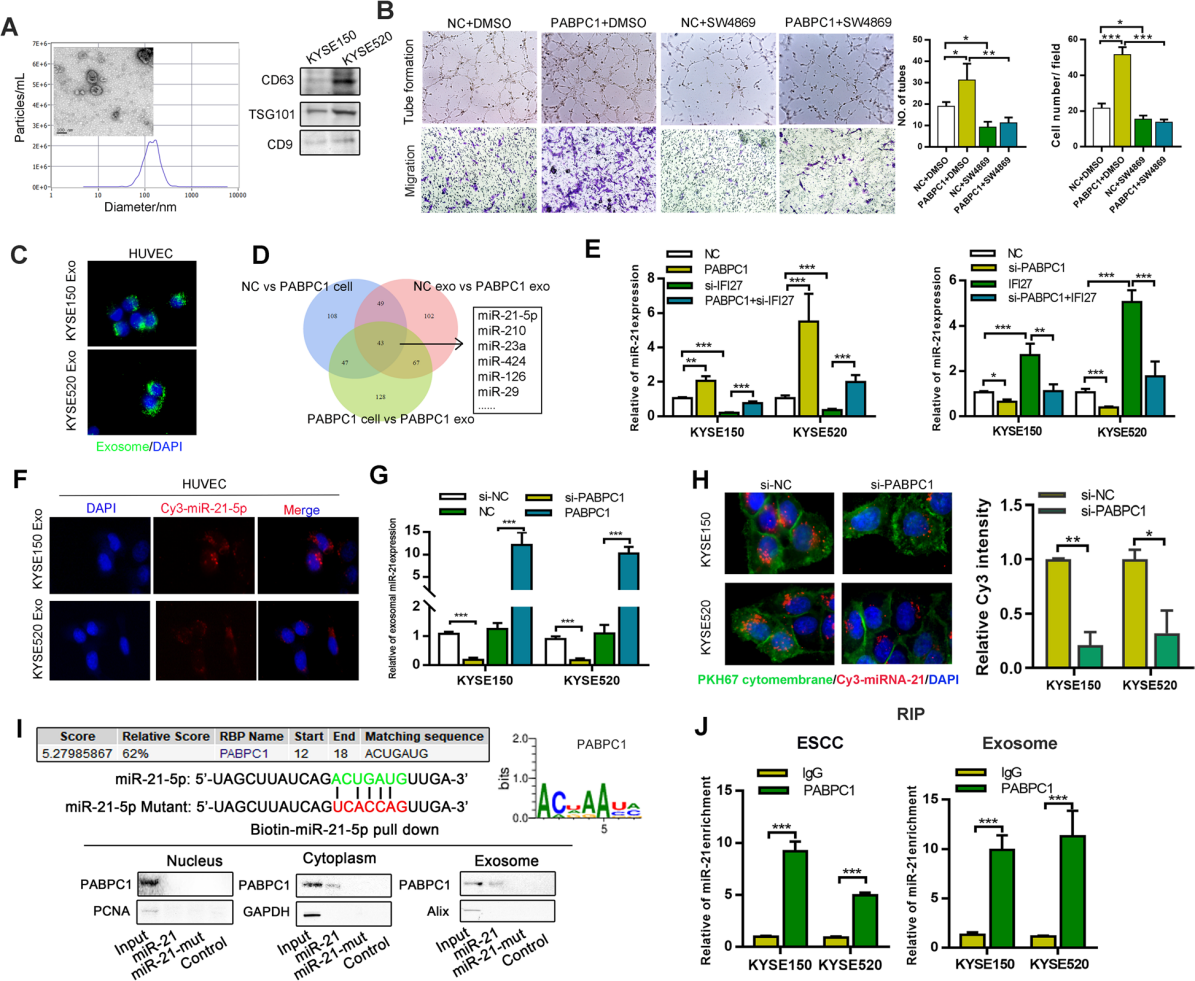
PABPC1/IFI27 regulate angiogenesis through exosome-transferred miR-21-5p regulation. **A** Representative images of exosomes by electron microscopy, NanoSight and western blotting (scale bars: 200 nm). **B** Transwell invasion and capillary tube formation assay of HUVECs treated with the indicated treatment and transfection (Scale bar: 50 µm). **C** HUVEC cells were incubated with PKH67-labeled exosomes from ESCCs for 24 h, and the green exosome signal was detected by confocal microscopy (scale bar, 20 µm).**D** MiRNA microarray was performed using PABPC1 or control transfected KYSE150 cells and cell-derived exosomes. **E** Expression of miR-21-5p was detected after the indicated transfection of KYSE150 and KYSE520 cells. **F** ESCCs transiently transfected with Cy3-tagged miR-21-5p were co-cultured with HUVEC cells for 48 h. Fluorescence microscopy was used to detect the red fluorescent signals in HUVECs (scale bar, 20 µm). **G** Expression of miR-21-5p was detected in ESCC-derived exosomes after the indicated transfection. **H** HUVECs were co-cultured with ESCCs concurrently transfected with Cy3-miR-21-5p and specific siRNAs targeting PABPC1 for 48 h. Fluorescence microscopy was used to detect fluorescent signals in HUVEC cells (scale bar, 10 µm). **I** Western blot analysis of PABPC1 expression in samples derived from miRNA pulldowns performed with nuclear, cytoplasmic, or exosomal ESCC lysates and the indicated biotinylated wildtype or mutated miR-21-5p; biotinylated poly(G) was used as a negative control. **J.** RIP assays with anti-PABPC1 antibody (or IgG as control) were performed on the cell or exosomal lysates from ESCCs. MiR-21-5p levels in immunoprecipitated samples were determined by qRT-PCR and were reported as percentages in respect to the input sample (% input). Data represent the mean ± SD of 3 separate determinations. *p < 0.05, **p < 0.01, ***p < 0.001 by Student’s t test

We then asked how PABPC1/IFI27 regulated angiogenesis in ESCC. We did not observe any significant changes in the expression levels of certain important angiogenesis-associated factors, including VEGFA, EGF, PDGFA, ANG1, and ANG2, after PABPC1 or IFI27 transfection (Figure S8A). Recently, it has been suggested that exosomes released from cancer cells can act as key factors to affect tumor angiogenesis and metastasis [22]. Thus, we isolated exosomes from ESCC cell culture medium and confirmed exosomes by western blotting, electron microscopy and nanoparticle tracking analysis (Fig. 6A). We further investigated whether PABPC1 could promote tumor angiogenesis through exosomes. We observed that exosomes derived from PABPC1-overexpressing ESCC cells conferred a substantial increase in the migration and tube formation of HUVECs when compared with exosomes from control cells, while exosomes from PABPC1-overexpressing ESCC cells, treated with the exosome inhibitor SW4869, largely restrained the migration and tube formation of HUVECs (Fig. 6B). In addition, our immunofluorescence assay demonstrated that PHK-67-stained exosomes were directly taken up by HUVECs *in vitro* (Fig. 6C). Collectively, these results suggest that PABPC1 exerts an angiogenic effect via ESCC cell exosomes.

Exosomal miRNA plays a key role in tumor angiogenesis [22]. Therefore, we used miRNA microarray analysis to explore the potential of exosomal miRNA in PABPC1-induced angiogenesis and identified miR-21-5p as the top candidate upregulated in exosomes from PABPC1-overexpressing cells, following comparison of exosomes from PABPC1-transfected cells and control cells (Fig. 6D and S8B, C). We initially confirmed that PABPC1 and IFI27 could mediate the expression of miR-21-5p in ESCC cells (Fig. 6E), and then we extracted exosomes from ESCC cells transfected with Cy3-labeled miR-21-5p mimics, and added the exosomes to HUVECs. Exosomal miR-21-5p could be taken by HUVECs and promote tube formation of HUVECs, indicating that ESCC cell-derived exosomal miR-21-5p could induce angiogenesis (Fig. 6F and S8D). Further investigation revealed that knockdown of PABPC1 decreased the level of miR-21-5p in ESCC cell-derived exosomes, as detected by qPCR (Fig. 6G). Additionally, knockdown of PABPC1 inhibited the process of exosomal miR-21-5p transfer from ESCC cells to HUVEC (Fig. 6H). To explore how PABPC1 mediated exosomal miR-21-5p, we analyzed the specific interaction between the miR-21-5p sequence and PABPC1 motifs (ACUGAUG) by using the database of RBP specificities (RBPDB, http://rbpdb.ccbr.utoronto.ca/; threshold 0.6). MiRNA pull-down assays revealed that there was significant binding between miR-21-5p and PABPC1 in the cytoplasm and in exosomes. However, this binding could be impaired by mutating the binding sequence of miR-21-5p (Fig. 6I). Thus, we speculated that PABPC1 may mediate the process of miR-21-5p packaging into exosomes. RIP assays further demonstrated that miR-21-5p was more enriched in the PABPC1 antibody group than in the IgG group, both in ESCC cells and their exosomal lysates (Fig. 6J). Therefore, we demonstrated that PABPC1 could stimulate angiogenesis by mediating miR-21-5p expression and promoting exosomal miR-21-5p packaging for release and targeting of endothelial cells.

### Exosomes miR-21-5p enhances tumor angiogenesis by suppressing CXCL10 in endothelial cells

**Fig. 7:**
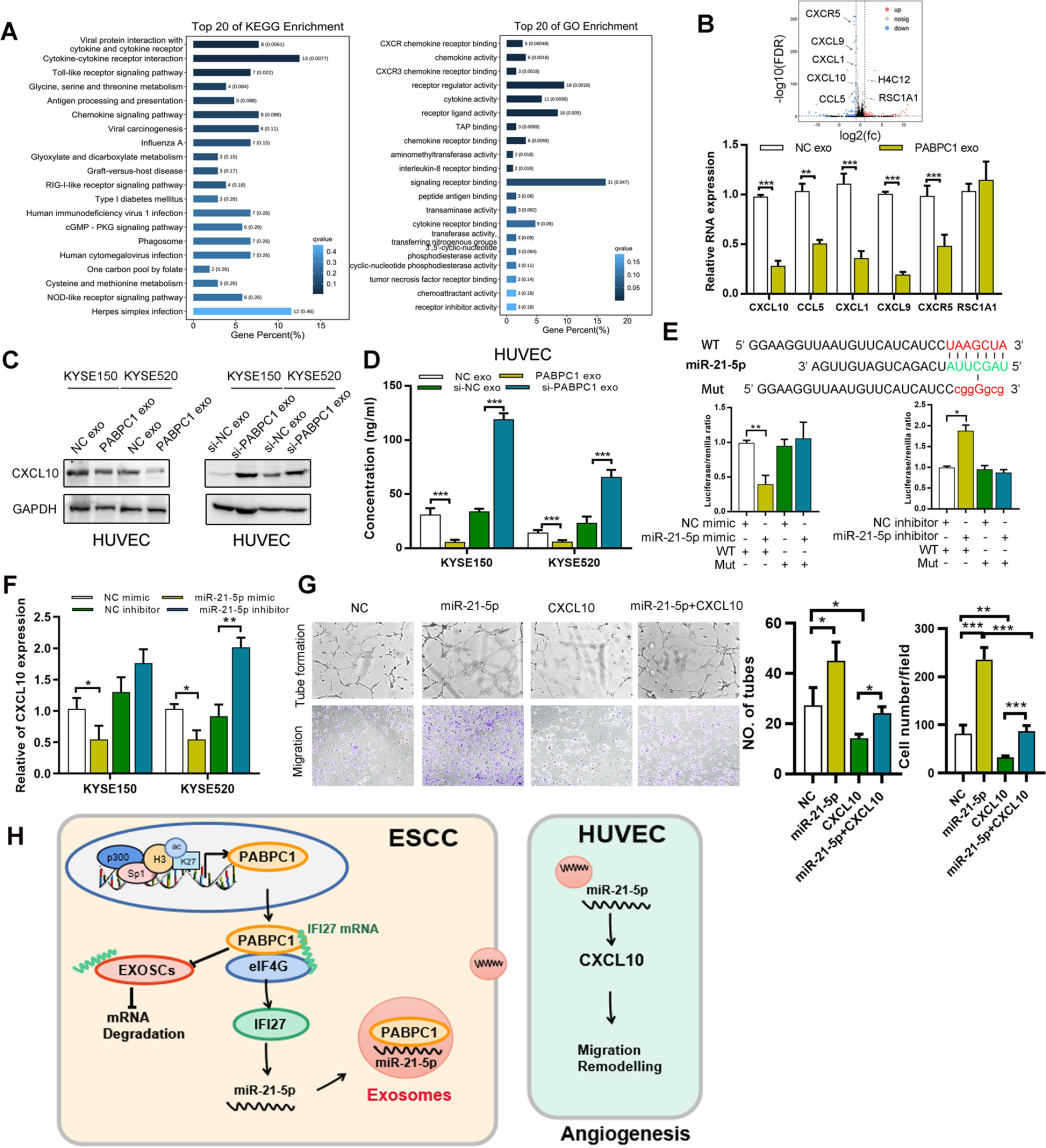
Exosomes enhance tumor angiogenesis by suppressing CXCL10 in endothelial cells. **A** RNA-seq was performed to detect the downstream pathways and differentially-expressed genes of HUVECs co-cultured with PABPC1-overexpressing ESCC cells. **B** The differentially-expressed genes are shown, and expression of selected genes was detected using qRT-PCR. **C** Detection of CXCL10 by western blotting. **D** Detection of CXCL10 by ELISA. **E** Predicted miR-21-5p target sequences in the 3’UTRs of CXCL10 mRNA. Relative CXCL10 reporter activities were detected in 293 T cells co-transfected with miR-21-5p and luciferase reporters. **F** Expression of miR-21-5p was detected after miR-21-5p transfection. **G** Transwell invasion and capillary tube formation assay of HUVECs treated with the indicated transfection (scale bar: 50 µm). **H** Proposed model illustrating the modulatory role of PABPC1 in promoting ESCC tumorigenesis. Data represent the mean ± SD of 3 separate determinations. *p < 0.05, **p < 0.01, ***p < 0.001 by Student’s t test

To analyze the mechanisms of regulated exosomes in controlling tumor angiogenesis, we performed RNA-seq to identify DEG profile and signaling pathways in HUVECs incubated with exosomes derived from PABPC1-overexpressing or control KYSE150 cells. KEGG and GO analyses of genes potentially regulated by exosomes revealed significant alternations in the cytokine-cytokine receptor signaling pathway (Fig. 7A). The top DEGs are shown in Fig. 7B. Numerous studies have shown that chemokine (C-X-C motif) ligand 10 (CXCL10), a target of miR-21-5p, is a key mediator in tumor angiostasis [23]. Western blotting and ELISA for CXCL10 showed a significant decrease in the expression of CXCL10 in HUVECs treated with exosomes derived from PABPC1-overexpressing cells (Fig. 7C, D). Bioinformatics predicted that CXCL10 was a putative direct miR-21-5p target. Using 3′ UTR luciferase reporter assays, we found that miR-21-5p overexpression inhibited luciferase activity in KYSE150 cells expressing wild-type CXCL10 3′ UTR reporters, whereas mutated miR-21-5p specifically abolished this suppression (Fig. 7E). Real-time PCR analysis revealed that a miR-21-5p mimic also inhibited CXCL10 expression in HUVEC cells, whereas a miR-21-5p inhibitor exhibited the opposite effect (Fig. 7F). Furthermore, the tube formation and cell migration of HUVECs treated with exosomal-miR-21-5p could be successfully halted by overexpressing CXCL10 (Fig. 7G).

## Discussion

In this study, we reported that PABPC1 expression is increased in ESCC, and is associated with histone acetylation in the PABPC1 promoter. We show ESCC patients with high PABPC1 expression have shorter survival compared with patients with low PABPC1 expression. Moreover, PABPC1 promotes ESCC cell growth and invasion via regulating IFI27. The mechanism by which this occurs involves PABPC1 binding eIF4G to block RNA exosome complex-mediated IFI27 mRNA degradation, and elevates miR-21-5p expression to increase miR-21-5p-containing exosomes that can target endothelial cells to increase angiogenesis (Fig. 7H).

The clinical significance of PABPC1 in ESCC was analyzed in samples from our own cohort and a public database (TCGA), which showed PABPC1 is upregulated in ESCC tissues compared with in case-matched normal epithelial tissues, which is consistent with several previous reports focusing on prostate cancer, HCC, and gastric cancer [24]. However, the mechanisms underlying the increased PABPC1 expression in cancer has not yet been clearly demonstrated. Dong et al. reported that PABPC1 expression could be mediated by SNHG14 by promoting histone acetylation in the SNHG14 promoter [25]. Histone acetylation is a major histone modification involved in the regulation of chromatin structure and transcription, and this process plays a key role in ESCC tumorigenesis [26]. Thus, using the ENCODE database, we found that H3K27ac is highly enriched at the PABPC1 promoter. The histone acetyltransferase p300 interacts with transcriptional coactivators to regulate acetyltransferase activities, thus promoting carcinogenesis[18]. Following experimental verification using ESCC tissues and cells, we identified the Sp1 transcription factor in combination with p300 could elevate histone acetylation at the PABPC1 promoter to elevate PABPC1 expression in ESCC. Sp1 and p300 expression are positively correlated with PABPC1 in ESCC tumors, further supporting our *in vitro* findings.

Furthermore, we found that PABPC1 promotes ESCC cell proliferation, migration, and invasion, and inhibits apoptosis, indicating that PABPC1 acts as a tumor promoter in ESCC cells. Similar findings have been reported in HCC and gastric cancer [9, 27]. However, the mechanisms by which PABPC1 promotes tumorigenesis in different cancer types are diverse. In hepatocellular carcinoma, PABPC1 interacts with AGO2 to augment miRNA repression by facilitating RISC binding and enhancing miRNA-mediated deadenylation, leading to the inhibition of tumor suppressor genes [9]. Zhu et al. found that PABPC1 upregulation plays a carcinogenic role by inhibiting mir-34c expression in gastric cancer cells [27]. In this study, we used RNA-seq to determine that the IFN pathway is a downstream pathway of PABPC1. The IFN pathway is a key mediator in tumor progression and exerts multiple biological effects, including antiviral and antitumor activities in patients with cancer and viral diseases [28]. Among these DEGs, we found IFI27, a key member of the IFN-α-induced protein family, to be upregulated by PABPC1. Rescue experiments revealed that PABPC1 promotes ESCC cell proliferation and invasion through upregulating IFI27.

We then explored how PABPC1 mediates IFI27 expression. Previous research has demonstrated that IFI27 protein is stabilized by EGF in keratinocytes [17]. However, according to our observations, protein stabilization did not contribute to high IFI27 expression in ESCC. In eukaryotic cells, mRNA homeostasis is achieved through a balance between mRNA synthesis and degradation. Since PABPC1 plays critical roles in mRNA translation, stabilization, and degradation, we predicted that PABPC1 could increase the stability of IFI27 mRNA. Indeed, we found that PABPC1 overexpression could enhance IFI27 mRNA stability by prolonging its half-life. Using mutant PABPC1 plasmids, we found that the PABPC1 could interact with eIF4G to protect IFI27 mRNA from degradation. Then, we further explored the detailed mechanisms of PABPC1-mediated IFI27 mRNA stabilization, which leads to ESCC tumorigenesis. In eukaryotic cells, the RNA exosome core contains a barrel-like structure (constituted by EXOSC 4 ~ 9) and a cap (constituted by EXOSC 1 ~ 3) [21]. The RNA exosome comprises a ring-like structure and two catalytic components, and plays a major role in various RNA processing and degradation pathways [29, 30]. EXOSCs are noncatalytic but are essential for the degradation and processing of target RNA, and EXOSC2 knockdown severely diminishes RNA exosome function [29]. In this study, we confirmed that EXOSC knockdown can markedly restore IFI27 mRNA expression, and PABPC1 could compete with EXOSCs to stabilize IFI27 mRNA.

Angiogenesis is defined as the formation of new blood vessels from preexisting vessels and has been characterized as an essential process for tumor cell proliferation and metastasis [31]. Exosomes derived from cancer cells contain a wide variety of miRNAs, and a large amount of evidence indicates that exosomal miRNAs are primary inducers of angiogenesis through activation of signaling pathways that trigger a network of signaling processes that promote endothelial cell growth, migration, and survival from pre-existing vasculature [26]. Our study shows that PABPC1 can increase expression of miR-21-5p, in ESCC cells, which is then encapsulated in ESCC cell-derived exosomes to target vessel endothelial cells and induce angiogenesis. Importantly, we further investigated how miR-21-5p is packaged into exosomes, showing that PABPC1 can bind miR-21-5p, through an ACUGAUG sequence, to direct miR-21-5p packaging into exosomes, similar to other studies showing involvement of other RBPs, such as hnRNPQ and hnRNPA2B1, in exosomal miRNA export [32, 33]. Previous studies showed the pro-angiogenic properties of endogenous miR-21-5p and cancer cell derived-miR-21-5p to be dependent on KRIT1 and Spry1 in vessel endothelial cells [34–36]. We show here that miR-21-5p-mediated suppression of CXCL10, a potent endogenous inhibitor of angiogenesis [23, 37], in HUVECs is in part responsible for tumor cell-induced angiogenesis and tumor growth in ESCC. The role of CXCL10 in inhibiting angiogenesis (angiostasis) has been extensively studied in various tissues and cancers, including the cornea, skeletal muscle cells and Kaposi sarcoma [37–39]. This newly discovered axis implicates miR-21-5p in multiple regulatory functions in angiogenesis. However, other functional components present in exosomes may also contribute to angiogenesis, and how miR-21-5p is packaged into exosomes has not been fully elucidated. Therefore, further research should be carried out to better illustrate these problems.

## Conclusion

In this study, we identify the role of PABPC1 in promoting ESCC tumorigenesis. Furthermore, we elucidate a novel mechanism by which PABPC1 enhances IFI27 mRNA stability by competing with the RNA exosome complex. PABPC1/IFI27 mediates miR-21-5p expression and promotes exosomal miR-21-5p packaging into exosomes that increase angiogenesis via transport of miR-21-5p to vascular endothelial cells, subsequently inhibiting vascular endothelial cell expression of CXCL10. These results increase our understanding of ESCC tumorigenesis, and thus provide a promising strategy for the treatment of ESCC.

## Supplementary Information

**Additional file 1.**

## Data Availability

The datasets used for the current study are available from the corresponding author on reasonable request. The authenticity of this article has been validated by uploading the key raw data onto the Research Data Deposit public platform (http://www.researchdata.org.cn/), the approval RDD number is RDDB2020000985.

## Abbreviations

5-aza: 5-Aza-2’-deoxycytidine
5-EU: 5-Ethynyl-uridine
Act D: Actinomycin D
CCK-8: Cell counting kit-8
CHX: Cycloheximide
ChIP: Chromatin immunoprecipitation
CI: Confidence interval
CIS: Carcinoma in situ
CXCL10: C-X-C motif chemokine 10
DAPI: 4’,6-Diamidino-2-phenylindole
DEG: Differentially expressed gene
DMEM: Dulbecco’ s modified eagle’ s medium
ERK: Extracellular regulated protein kinase
ESCC: Esophageal squamous cell carcinoma
FBS: Fetal bovine serum
GAPDH: Glyceraldehyde-3-phosphate dehydrogenase
GO: Gene Ontology
HDAC: Histone deacetylase
HR: Hazard ratio
HUVEC: Human umbilical vein endothelial cell
IHC: Immunohistochemistry
IFI27: IFN-α inducible protein 27
IFN: Interferon
IP: Immunoprecipitation
KO: Knockout
NC: Negative control
MG132: Carbobenzoxy-Leu-Leu-leucinal
MVD: Microvessel density
NF-kB: Nuclear factor kappa B
NSCLC: Non-small-cell lung cancer
PBS: Phosphate-buffered saline
qRT-PCR: Quantitative reverse transcription polymerase chain reaction
STAT3: Signal transducer and activator of transcription 3
3’ UTR: 3’ Untranslated region
RPMI: Roswell Park Memorial Institute

## References

[1] Kashyap MK, Abdel-Rahman O (2018). Expression, regulation and targeting of receptor tyrosine kinases in esophageal squamous cell carcinoma. Mol Cancer.

[2] Lin EW, Karakasheva TA, Hicks PD, Bass AJ, Rustgi AK (2016). The tumor microenvironment in esophageal cancer. Oncogene.

[3] Schiffmann LM, Plum PS, Fuchs HF, Babic B, Bruns CJ, Schmidt T (2021). Tumor Microenvironment of Esophageal Cancer. Cancers..

[4] Jing Z, Chen K, Gong L (2021). The Significance of Exosomes in Pathogenesis, Diagnosis, and Treatment of Esophageal Cancer. Int J Nanomed.

[5] Mangus DA, Evans MC, Jacobson A (2003). Poly(A)-binding proteins: multifunctional scaffolds for the post-transcriptional control of gene expression. Genome Biol.

[6] Uren PJ, Vo DT, de Araujo PR, Potschke R, Burns SC, Bahrami-Samani E (2015). RNA-Binding Protein Musashi1 Is a Central Regulator of Adhesion Pathways in Glioblastoma. Mol Cell Biol.

[7] Ozturk S, Uysal F (2017). Poly(A)-binding proteins are required for translational regulation in vertebrate oocytes and early embryos. Reprod Fertil Dev.

[8] Chorghade S, Seimetz J, Emmons R, Yang J, Bresson SM, Lisio M (2017). Poly(A) tail length regulates PABPC1 expression to tune translation in the heart. Elife..

[9] Zhang H, Sheng C, Yin Y, Wen S, Yang G, Cheng Z (2015). PABPC1 interacts with AGO2 and is responsible for the microRNA mediated gene silencing in high grade hepatocellular carcinoma. Cancer Lett.

[10] Wang Q, Wang Z, Bao Z, Zhang C, Wang Z, Jiang T (2020). PABPC1 relevant bioinformatic profiling and prognostic value in gliomas. Future Oncol.

[11] Dizin E, Gressier C, Magnard C, Ray H, Decimo D, Ohlmann T (2006). BRCA1 interacts with poly(A)-binding protein: implication of BRCA1 in translation regulation. J Biol Chem.

[12] Takashima N, Ishiguro H, Kuwabara Y, Kimura M, Haruki N, Ando T (2006). Expression and prognostic roles of PABPC1 in esophageal cancer: correlation with tumor progression and postoperative survival. Oncol Rep.

[13] Suomela S, Cao L, Bowcock A, Saarialho-Kere U (2004). Interferon alpha-inducible protein 27 (IFI27) is upregulated in psoriatic skin and certain epithelial cancers. J Invest Dermatol.

[14] Chiang KC, Huang ST, Wu RC, Huang SC, Yeh TS, Chen MH (2019). Interferon alpha-inducible protein 27 is an oncogene and highly expressed in cholangiocarcinoma patients with poor survival. Cancer management and research.

[15] Li S, Xie Y, Zhang W, Gao J, Wang M, Zheng G (2015). Interferon alpha-inducible protein 27 promotes epithelial-mesenchymal transition and induces ovarian tumorigenicity and stemness. J Surg Res.

[16] Wang H, Qiu X, Lin S, Chen X, Wang T, Liao T (2018). Knockdown of IFI27 inhibits cell proliferation and invasion in oral squamous cell carcinoma. World journal of surgical oncology.

[17] Hsieh WL, Huang YH, Wang TM, Ming YC, Tsai CN, Pang JH (2015). IFI27, a novel epidermal growth factor-stabilized protein, is functionally involved in proliferation and cell cycling of human epidermal keratinocytes. Cell Prolif.

[18] Zhang Y, Liu Z, Yang X, Lu W, Chen Y, Lin Y (2021). H3K27 acetylation activated-COL6A1 promotes osteosarcoma lung metastasis by repressing STAT1 and activating pulmonary cancer-associated fibroblasts. Theranostics.

[19] Zhang Y, Chen Y, Yun H, Liu Z, Su M, Lai R (2017). STAT1beta enhances STAT1 function by protecting STAT1alpha from degradation in esophageal squamous cell carcinoma. Cell Death Dis.

[20] Huntzinger E, Braun JE, Heimstadt S, Zekri L, Izaurralde E (2010). Two PABPC1-binding sites in GW182 proteins promote miRNA-mediated gene silencing. EMBO J.

[21] Makino DL, Halbach F, Conti E (2013). The RNA exosome and proteasome: common principles of degradation control. Nat Rev Mol Cell Biol.

[22] Wu F, Li F, Lin X, Xu F, Cui RR, Zhong JY (2019). Exosomes increased angiogenesis in papillary thyroid cancer microenvironment. Endocr Relat Cancer.

[23] Liu M, Guo S, Hibbert JM, Jain V, Singh N, Wilson NO (2011). CXCL10/IP-10 in infectious diseases pathogenesis and potential therapeutic implications. Cytokine Growth Factor Rev.

[24] Eisermann K, Dar JA, Dong J, Wang D, Masoodi KZ, Wang Z (2015). Poly (A) binding protein cytoplasmic 1 is a novel co-regulator of the androgen receptor. PloS One.

[25] Dong H, Wang W, Mo S, Liu Q, Chen X, Chen R (2018). Long non-coding RNA SNHG14 induces trastuzumab resistance of breast cancer via regulating PABPC1 expression through H3K27 acetylation. J Cell Mol Med.

[26] Guo P, Chen W, Li H, Li M, Li L (2018). The histone acetylation modifications of breast cancer and their therapeutic implications. Pathol Oncol Res.

[27] Zhu J, Ding H, Wang X, Lu Q (2015). PABPC1 exerts carcinogenesis in gastric carcinoma by targeting miR-34c. Int J Clin Exp Pathol.

[28] Ahmed D, Cassol E (2017). Role of cellular metabolism in regulating type I interferon responses: implications for tumour immunology and treatment. Cancer Lett.

[29] Morton DJ, Kuiper EG, Jones SK, Leung SW, Corbett AH, Fasken MB (2018). The RNA exosome and RNA exosome-linked disease. RNA.

[30] Zhang W, Zhu J, He X, Liu X, Li J, Li W (2019). Exosome complex genes mediate RNA degradation and predict survival in mantle cell lymphoma. Oncol Lett.

[31] Li S, Xu HX, Wu CT, Wang WQ, Jin W, Gao HL (2019). Angiogenesis in pancreatic cancer: current research status and clinical implications. Angiogenesis.

[32] Si Y, Liu F, Wang D, Fang C, Tang X, Guo B (2021). Exosomal transfer of miR-185 Is controlled by hnRNPA2B1 and impairs re-endothelialization after vascular injury. Front Cell Dev Biol.

[33] Santangelo L, Giurato G, Cicchini C, Montaldo C, Mancone C, Tarallo R (2016). The RNA-binding protein SYNCRIP is a component of the hepatocyte exosomal machinery controlling MicroRNA sorting. Cell Rep.

[34] He Q, Ye A, Ye W, Liao X, Qin G, Xu Y (2021). Cancer-secreted exosomal miR-21-5p induces angiogenesis and vascular permeability by targeting KRIT1. Cell Death Dis.

[35] Liu LZ, Li C, Chen Q, Jing Y, Carpenter R, Jiang Y (2011). MiR-21 induced angiogenesis through AKT and ERK activation and HIF-1alpha expression. PloS One.

[36] Ma S, Zhang A, Li X, Zhang S, Liu S, Zhao H (2020). MiR-21-5p regulates extracellular matrix degradation and angiogenesis in TMJOA by targeting Spry1. Arthritis Res Ther.

[37] Gao N, Liu X, Wu J, Li J, Dong C, Wu X (2017). CXCL10 suppression of hem- and lymph-angiogenesis in inflamed corneas through MMP13. Angiogenesis.

[38] Dinsart C, Pervolaraki K, Stroh-Dege A, Lavie M, Ronsse I, Rommelaere J (2017). Recombinant parvoviruses armed to deliver CXCL4L1 and CXCL10 are impaired in their antiangiogenic and antitumoral effects in a Kaposi sarcoma tumor model due to the chemokines' interference with the virus cycle. Hum Gene Ther.

[39] Ishiuchi Y, Sato H, Tsujimura K, Kawaguchi H, Matsuwaki T, Yamanouchi K (2018). Skeletal muscle cell contraction reduces a novel myokine, chemokine (C-X-C motif) ligand 10 (CXCL10): potential roles in exercise-regulated angiogenesis. Biosci Biotechnol Biochem.

