# Supplementary material for: PABPC1-induced stabilization of IFI27 mRNA promotes angiogenesis and malignant progression in esophageal squamous cell carcinoma through exosomal miRNA-21-5p
Source: J Exp Clin Cancer Res. 2022 Mar 28;41:111. doi: 10.1186/s13046-022-02339-9 (PMC8962095; doi:10.1186/s13046-022-02339-9)
Supplement: Supplementary file 1 — Additional file 1. [file 13046_2022_2339_MOESM1_ESM.zip › Supplemental material.docx]

**Supplemental table 1**. **Primers**

| **Gene** | **F** | **R** |
| --- | --- | --- |
| **IFI27** | CTTCACTGCGGCGGGAATC | CCAGGATGAACTTGGTCAATCC |
| **PABPC1** | AGCAAATGTTGGGTGAACGG | ACCGGTGGCACTGTTAACTG |
| **MMP10** | ACTCATTCACAGAGCTCGCC | AGCAGGATCACACTTGGCTG |
| **GNGT1** | CACTGGTAAAGGGCATCCCA | ATGGGTTACCACACCCCAAT |
| **IFI27 promoter primer 1** | GGGTCACTGGAGTCTCTGAC | TGGTTCTCTTCTCTGCAGGG |
| **IFI27 promoter primer 2** | GCTGTCATTGCGAGGTTCTA | CCTCTGGAGATGCAGAATTTGG |
| **GSDMC** | TCCATGTTGGAACGCATTAGC | CAAACTGACGTAATTTGGTGGC |
| **ONECUT1** | GAACATGGGAAGGATAGAGGCA | GTAGAGTTCGACGCTGGACAT |
| **OAS2** | CTCAGAAGCTGGGTTGGTTTAT | ACCATCTCGTCGATCAGTG |
| **SPOCK1** | CCCAACCACGGCAATTTCCTA | ATCGTCTCGAAAGCGGTTCC |
| **VEGFA** | TTGCCTTGCTGCTCTACCTCCA | GATGGCAGTAGCTGCGCTGATA |
| **JAG** | GTCCATGCAGAACGTGAACG | GCGGGACTGATACTCCTTGA |
| **NRP1** | GGCGCTTTTCGCAACGATAAA | TCGCATTTTTCACTTGGGTGAT |
| **STAB1** | AACCACGTTTGTCACTCATGT | CGGCAGTCCTGGGTTATCTG |
| **FGF2** | AGAAGAGCGACCCTCACATCA | CGGTTAGCACACACTCCTTTG |
| **ANGPT2** | AACTTTCGGAAGAGCATGGAC | CGAGTCATCGTATTCGAGCGG |
| **ANPEP** | GACCAAAGTAAAGCGTGGAATCG | TCTCAGCGTCACCCGGTAG |
| **VEGFC** | GAGGAGCAGTTACGGTCTGTG | TCCTTTCCTTAGCTGACACTTGT |
| **PDGFA** | GCAAGACCAGGACGGTCATTT | GGCACTTGACACTGCTCGT |
| **MiR-21-5p** | AACACCAGTCGATGGGCTGT | AGTGCAGGGTCCGAGGTATT |
| **MiR-210** | CTGTGCCTGGGCAGCG | AGTGCAGGGTCCGAGGTATT |
| **MiR-23a** | CATTGCCAGGGATTTCCAA | AGTGCAGGGTCCGAGGTATT |
| **MiR-424** | CCTCGTGGGGAAGGTAGAAG | AGTGCAGGGTCCGAGGTATT |
| **MiR-296** | GGGTGGAGGCTCTCCTGAAG | AGTGCAGGGTCCGAGGTATT |
| **MiR-126** | ACAGTTCTCTCGTACCGTGAGTAAT | AGTGCAGGGTCCGAGGTATT |
| **MiR-29** | TAGCACCATCTGAAATCGGTTA | AGTGCAGGGTCCGAGGTATT |
|  |  |  |
|  |  |  |
|  |  |  |
|  |  |  |

**Supplemental Figure 1**. **A**. The expression of PABPC1 was higher in ESCC (T) than normal tissues (N) in 15 cases, equal in 5 cases and lower in 3 cases. **B.** The immunohistochemistry score of PABPC1 in normal and ESCC. **B.** The expression of PABPC1 was detected in the cytoplasm and nucleus of ESCC cells KYSE150 and KYSE520. **C.** The survival of ESCC patients from public database TCGA was analyzed by Kaplan-meier curve. **D.** Forest plot showed the association between clinical parameters, PABPC1 expression and ESCC survival using univariate and multivariate analyses. (HR, hazard ratio; CI, confidence interval). *p < 0.05, **p < 0.01, ***p < 0.001 by Student’s t test.

**Supplemental Figure 2**. **A**. The genome bioinformatics analysis showed that H3K27ac was enriched at the promoter of PABPC1 by using UCSC Genome Bioinformatics Site (http://genome.ucsc.edu/). **B**. The expression of PABPC1 was detected by western blot and qRT-PCR after transfecting different dose of p300 plasmid. **C**. The expression of PABPC1 was detected by western blot and qRT-PCR after transfecting different dose of sp1 plasmid. Data represent the mean±SD of 3 separate determinations. *p < 0.05, **p < 0.01, ***p < 0.001 by Student’s t test.

**Supplemental Figure 3**. **A.** CCK8 assay was performed on control and PABPC1-overexpressing and PABPC1 knockdown ESCC cells. **B.** Colony formation assay was performed on control and PABPC1-overexpressing and knockdown ESCC cells. **C**. Annexin V-PI assay was performed to detect the apoptosis rate in PABPC1-overexpressing and knockdown ESCC cells. **D** Western blot was performed to detect the expression of PARP and caspase 9 on PABPC1-overexpressing and knockdown ESCC cell lines. Data represent the mean±SD of 3 separate determinations. *p < 0.05, **p < 0.01, ***p < 0.001 by Student’s t test.

**Supplemental Figure 4. A.** The indicated gene mRNA expression was detected by qRT-PCR after transfecting PABPC1. **B**. Annexin V-PI assay was performed to detect the apoptosis rate on indicated transfected ESCC cells. **C.** The expression of PARP and caspase 9 expression was detected in ESCC cell lines with indicated transfection. **D**. Colony formation assay was performed on indicated transfected ESCC KYSE150 cells. **E.** CCK8 was performed to detect the proliferation rate of indicated transfected ESCC KYSE150 cells. **F.** Transwell assay was performed on control and indicated transfect cells to detect the invasion ability of ESCC cells. **G.** The expression of STAT3, NF-kB and ERK expression was detected in ESCC cell lines with indicated transfection. **H**. Representative IHC image of nude mouse tumor tissues for the expression of Ki-67, IFI27 and caspase 3 from KYSE150 cells treated as indicated (Scale bars: 200 μm). Data represent the mean±SD of 3 separate determinations. *p < 0.05, **p < 0.01, ***p < 0.001 by Student’s t test.

**Supplemental Figure 5. A.** The expression of IFI27 in different types of cancer in GEPIA database (http://gepia.cancer-pku.cn/). **B.** The expression of IFI27 was explored in public database (GSE20347, GSE23400). **C**. The expression of IFI27 was detected in normal esophageal epithelial and ESCC by immunohistochemistry (n=190, Scale bar: 200 μm, 100 μm). **D**. The expression of IFI27 in normal esophageal tissues and case matched ESCC was detected by western blot (n= 24). **E.** Comparison of overall survival of ESCC patients with high and low IFI27 protein expression was detected by Kaplan-Meier curve in our case cohort and TCGA database. Data represent the mean±SD of 3 separate determinations. *p < 0.05, **p < 0.01, ***p < 0.001 by Student’s t test.

**Supplemental Figure 6. A.** The expression of IFI27 was detected after treating with indicated dose of MG132 by western blot in ESCC cell lines. **B.** The IFI27 mRNA synthesis efficiency was detected by nascent RNA capture assays in the cell with PABPC1 transfection or control. **C.** The transcription activity of IFI27 was detected by dual-luciferase reporter assay. **D**. The poly A tail length of IFI27 mRNA was detected after PABPC1 transfected in KYSE150 and KYSE520. Data represent the mean±SD of 3 separate determinations. **E.** The expression of IFI27 mRNA was detected after si-PABPC1 transfection and treated with the transcription inhibitor actinomycin D (5 mg/mL) in KYSE150 and KYSE520. **F.** The interaction of PABPC1 and IFI27 mRNA was detected by RNA biotin pulldown assay in KYSE150 and KYSE520. **G.** The interaction of PABPC1 and eIF4G was detected by co-immunoprecipitation after si-eIF4G transfection in KYSE150 and KYSE520. **H.** The interaction of PABPC1 and IFI27 mRNA was detected by RNA biotin pulldown assay after si-eIF4G transfection in KYSE150 and KYSE520. **I.** The interaction of PABPC1 and IFI27 mRNA was detected by RIP after si-eIF4G transfection in KYSE150 and KYSE520. **J.** The binding of HA-tagged-eIF4G and flag-tagged RRM1-4 or MLLE were detected by co-immunoprecipitation in HEK293T cell. **K.** The binding of flag-tagged wide type PABPC1, PABPC1 ΔRRM1 and HA-tagged-eIF4G was detected by co-immunoprecipitation in HEK293T cell. **L.** The expression of IFI27 mRNA was detected after PABPC1 ΔRRM1 transfection and treated with the transcription inhibitor actinomycin D (5 mg/mL) in KYSE150 and KYSE520. **M.** The binding of PABPC1 and eIF4G was detected by co-immunoprecipitation after knockdown IFI27 in KYSE150 and KYSE520.Data represent the mean±SD of 3 separate determinations. *p < 0.05, **p < 0.01, ***p < 0.001 by Student’s t test.

**Supplemental Figure 7**. **A**. Western blot and qRT-PCR analysis demonstrating IFI27 protein and mRNA levels following knockdown of EXOSC2 in KYSE150 and KYSE520 cells with two separate siRNAs. **B**. qRT-PCR analysis of IFI27 mRNA levels in KYSE150 and KYSE520 cells infected with the indicated siRNAs. **C**. RIP assay demonstrating that knockdown of PABPC1 increases the binding affinity of EXOSC2 with IFI27 mRNA, while knockdown of EXOSC2 increases the binding affinity of PABPC1 with IFI27 mRNA. **D**. The expression of IFI27 was detected by western blot and qRT-PCR in KYSE150 cells after EXOCS4 knockdown. **E**. RIP assay demonstrating that knockdown of PABPC1 increases the binding affinity of EXOSC4 with IFI27 mRNA, while knockdown of EXOSC4 increases the binding affinity of PABPC1 with IFI27 mRNA. **F.** The mRNA expression of IFI27 was detected by qRT-PCR in the KYSE150 and KYSE520 with indicated treatment. Data represent the mean±SD of 3 separate determinations. *p < 0.05, **p < 0.01, ***p < 0.001 by Student’s t test.

**Supplemental Figure 8. A.** The RNA expression of angiogenesis factors was detected after PABPC1 or IFI27 transfection in ESCC cells KYSE150. **B.** The expression of miRNAs was detected after PABPC1 or si-PABPC1 transfection in ESCC cells KYSE150. **C.** The expression of miR-21-5p was detected after PABPC1 or si-PABPC1 transfection in ESCC cells KYSE150 and KYSE520. **D.** Capillary tube formation assay of HUVECs treated with the exosome derived from miR-21-5p or control transfected KYSE150 and KYSE520 cells. Data represent the mean±SD of 3 separate determinations. *p < 0.05, **p < 0.01, ***p < 0.001 by Student’s t test.
