# Supplementary figures and images for: PABPC1-induced stabilization of IFI27 mRNA promotes angiogenesis and malignant progression in esophageal squamous cell carcinoma through exosomal miRNA-21-5p
Source: J Exp Clin Cancer Res. 2022 Mar 28;41:111. doi: 10.1186/s13046-022-02339-9 (PMC8962095; doi:10.1186/s13046-022-02339-9)

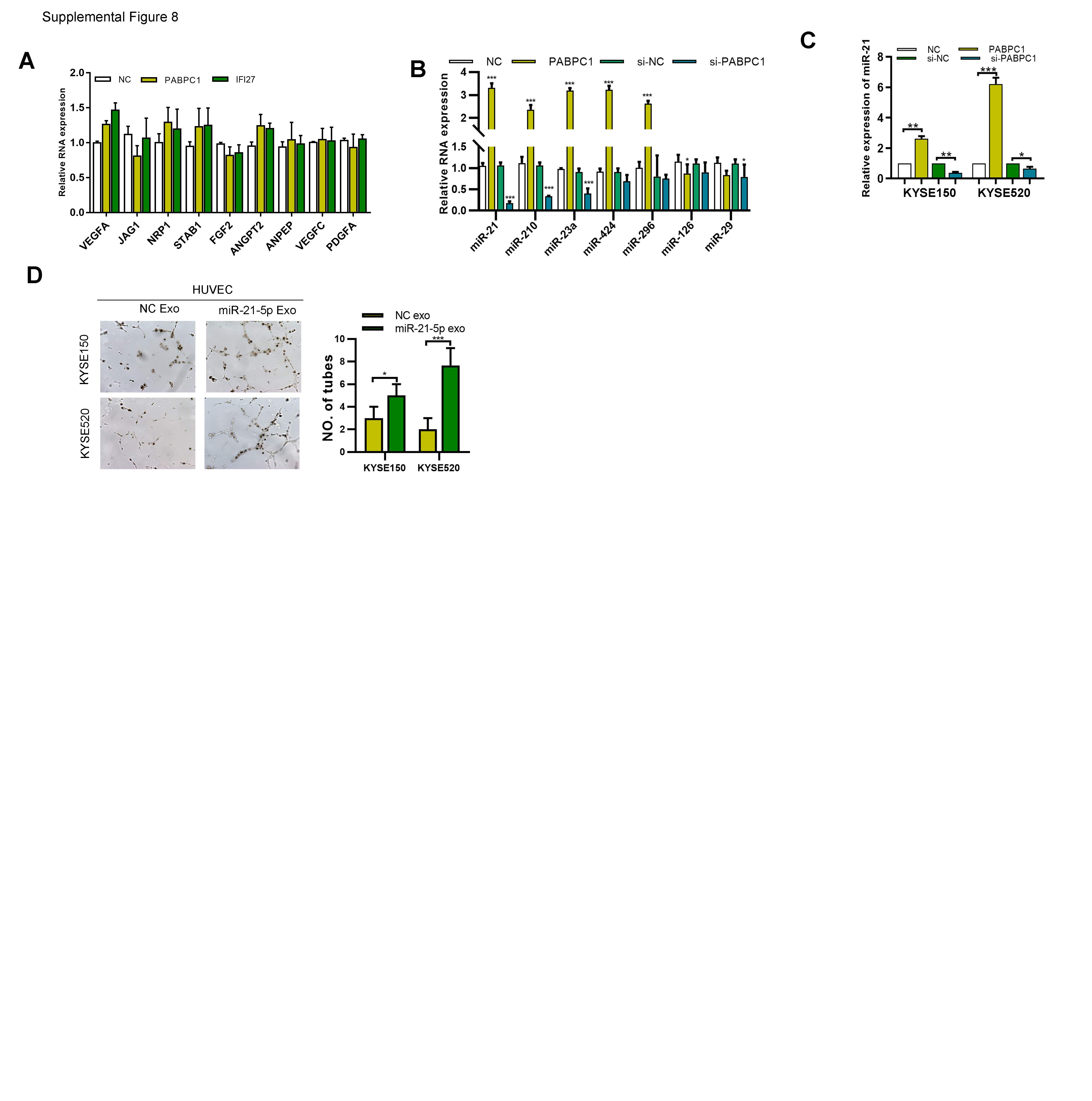

Supplement: Supplementary file 1 — Additional file 1. [file 13046_2022_2339_MOESM1_ESM.zip › renamed_3222a.tif]

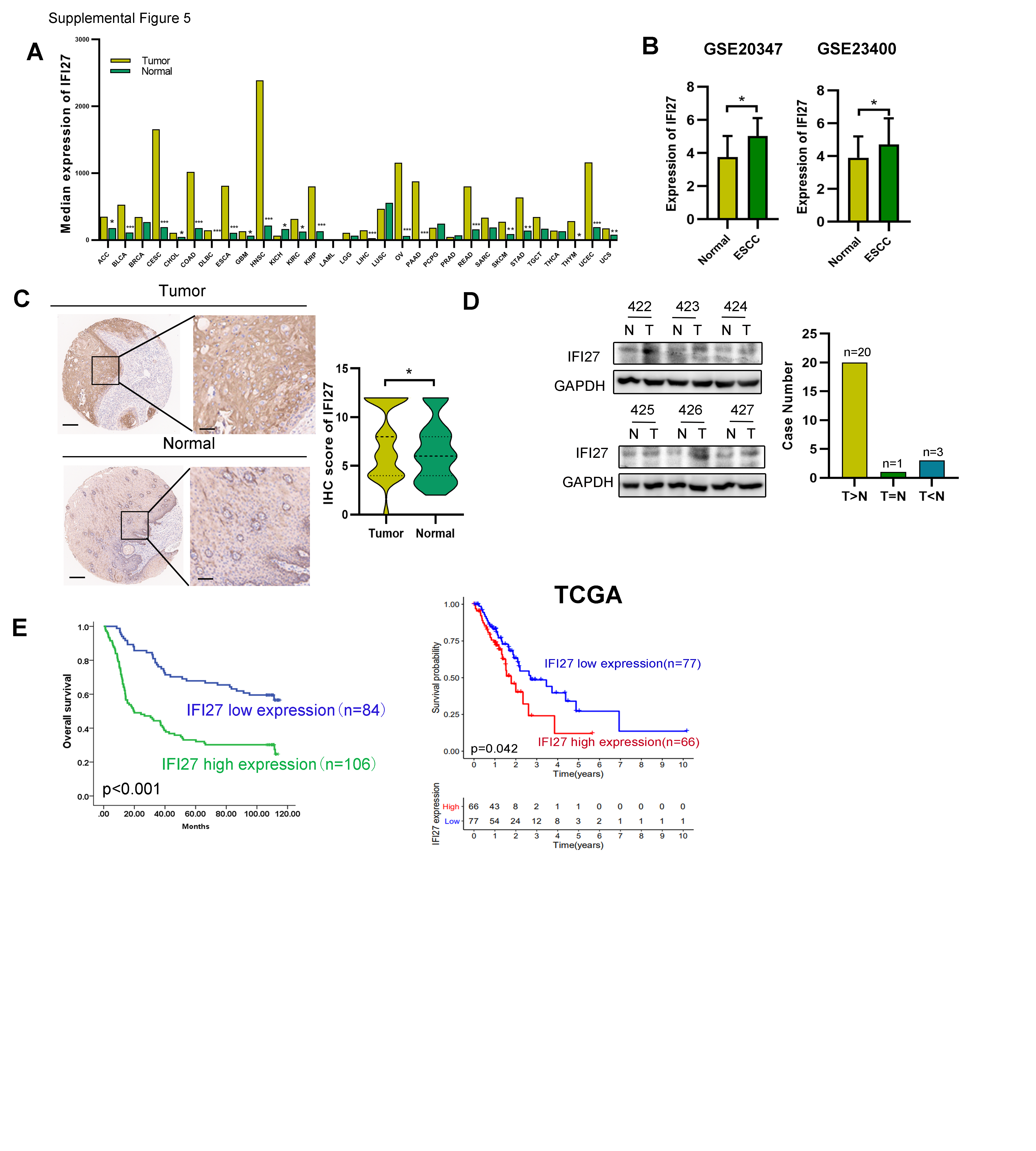

Supplement: Supplementary file 1 — Additional file 1. [file 13046_2022_2339_MOESM1_ESM.zip › renamed_68411.tif]

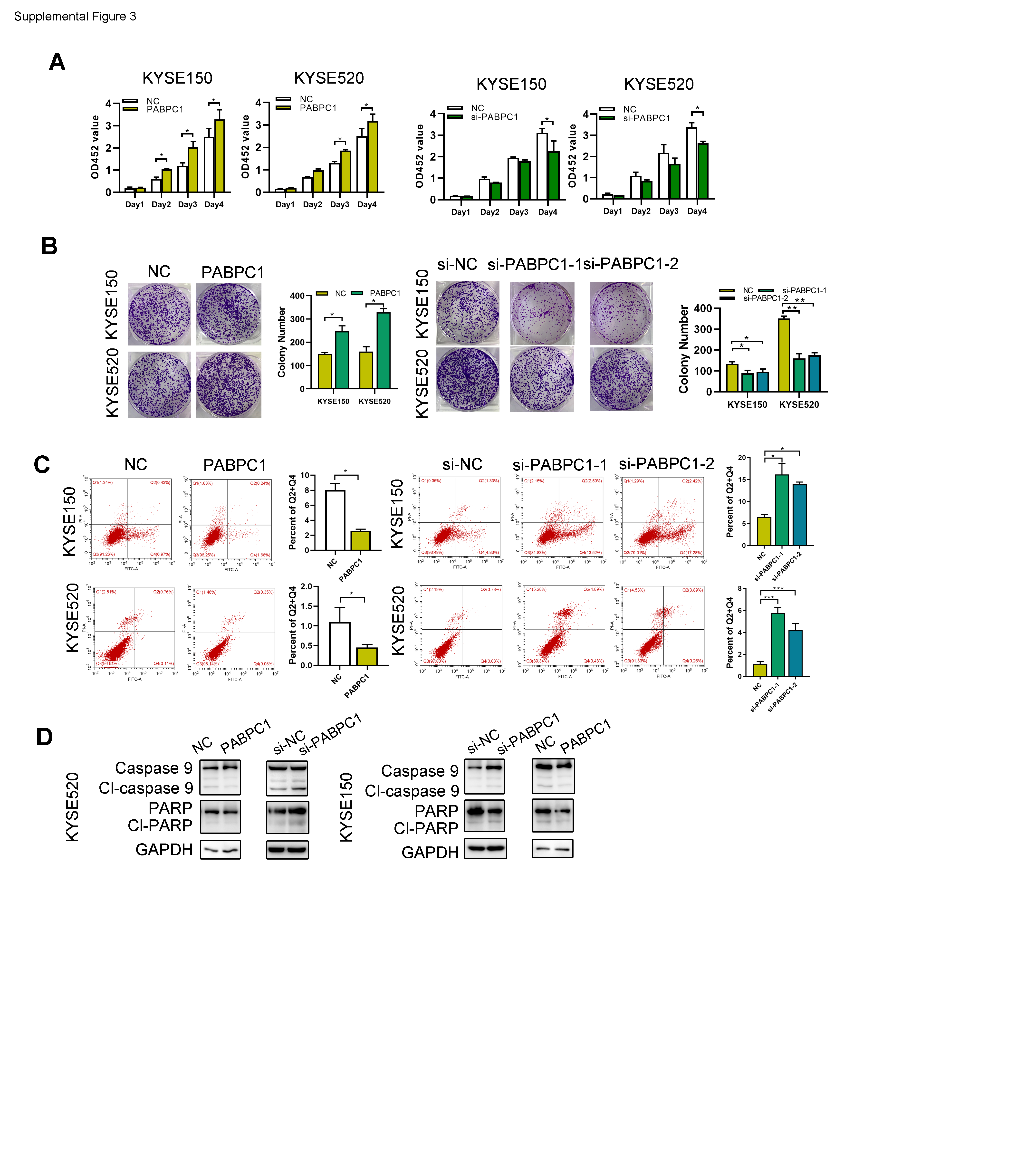

Supplement: Supplementary file 1 — Additional file 1. [file 13046_2022_2339_MOESM1_ESM.zip › renamed_9248f.tif]

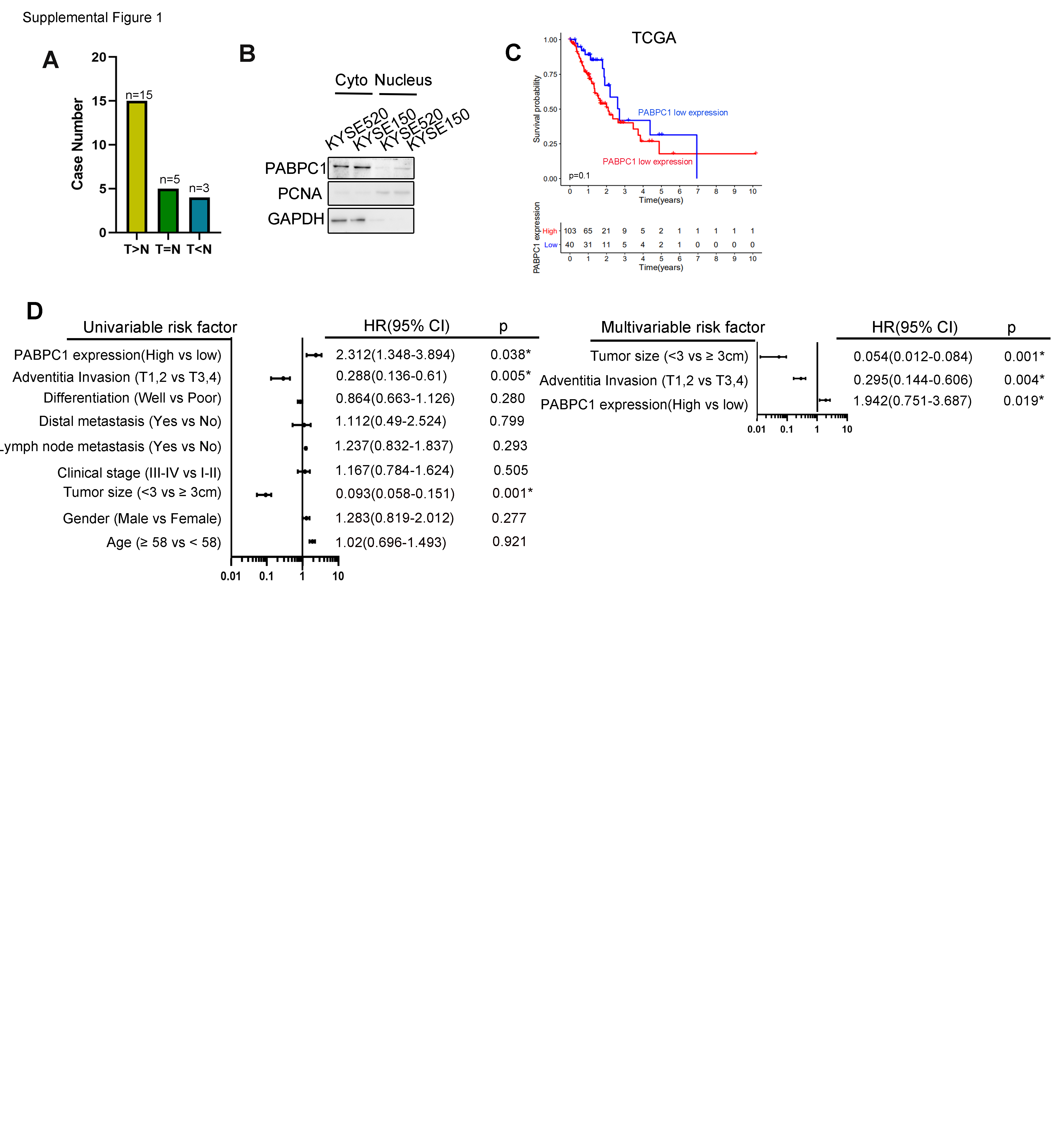

Supplement: Supplementary file 1 — Additional file 1. [file 13046_2022_2339_MOESM1_ESM.zip › renamed_f4eeb.tif]

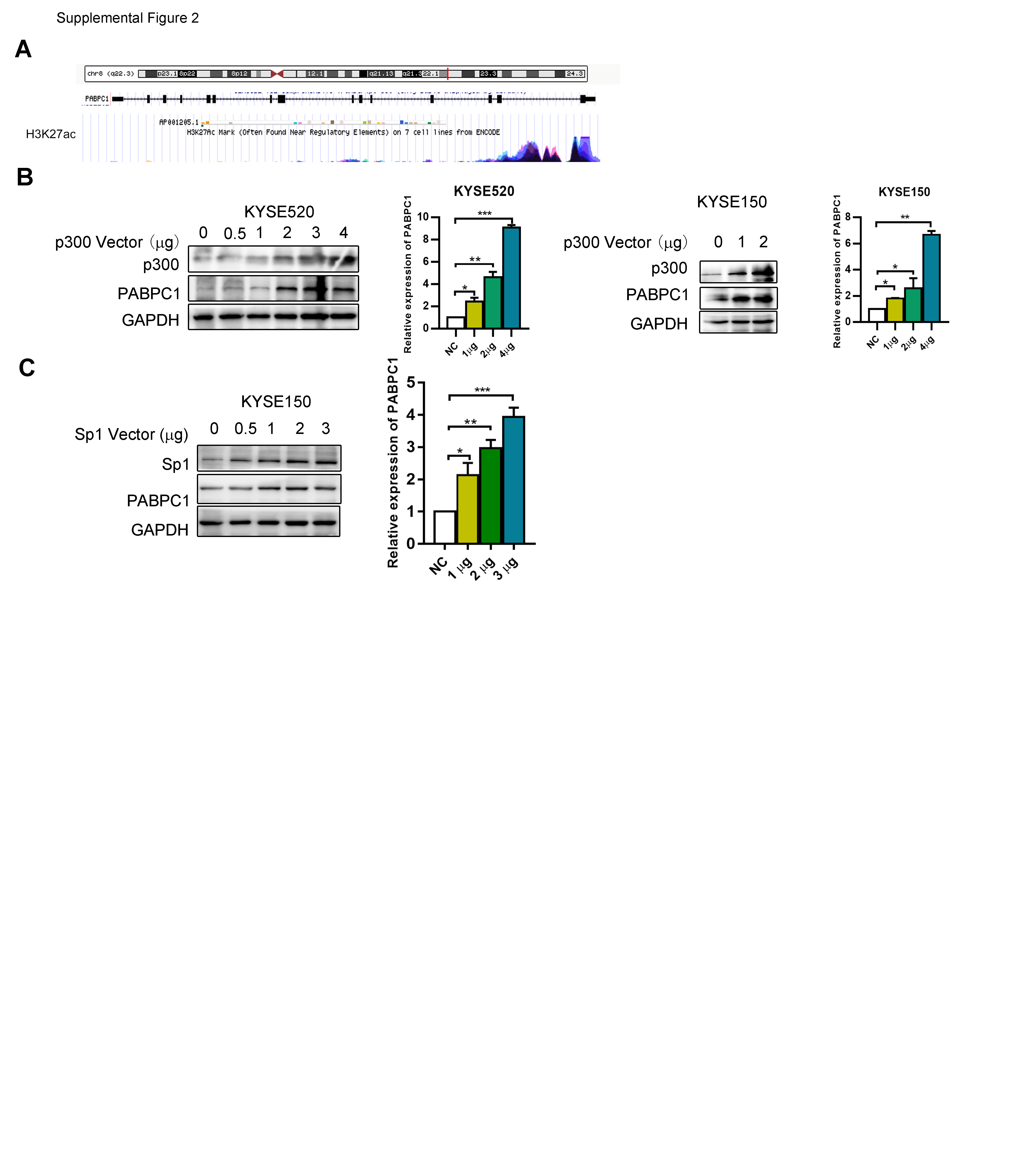

Supplement: Supplementary file 1 — Additional file 1. [file 13046_2022_2339_MOESM1_ESM.zip › SUPPLEMENTAL figure 2-H3K27.tif]

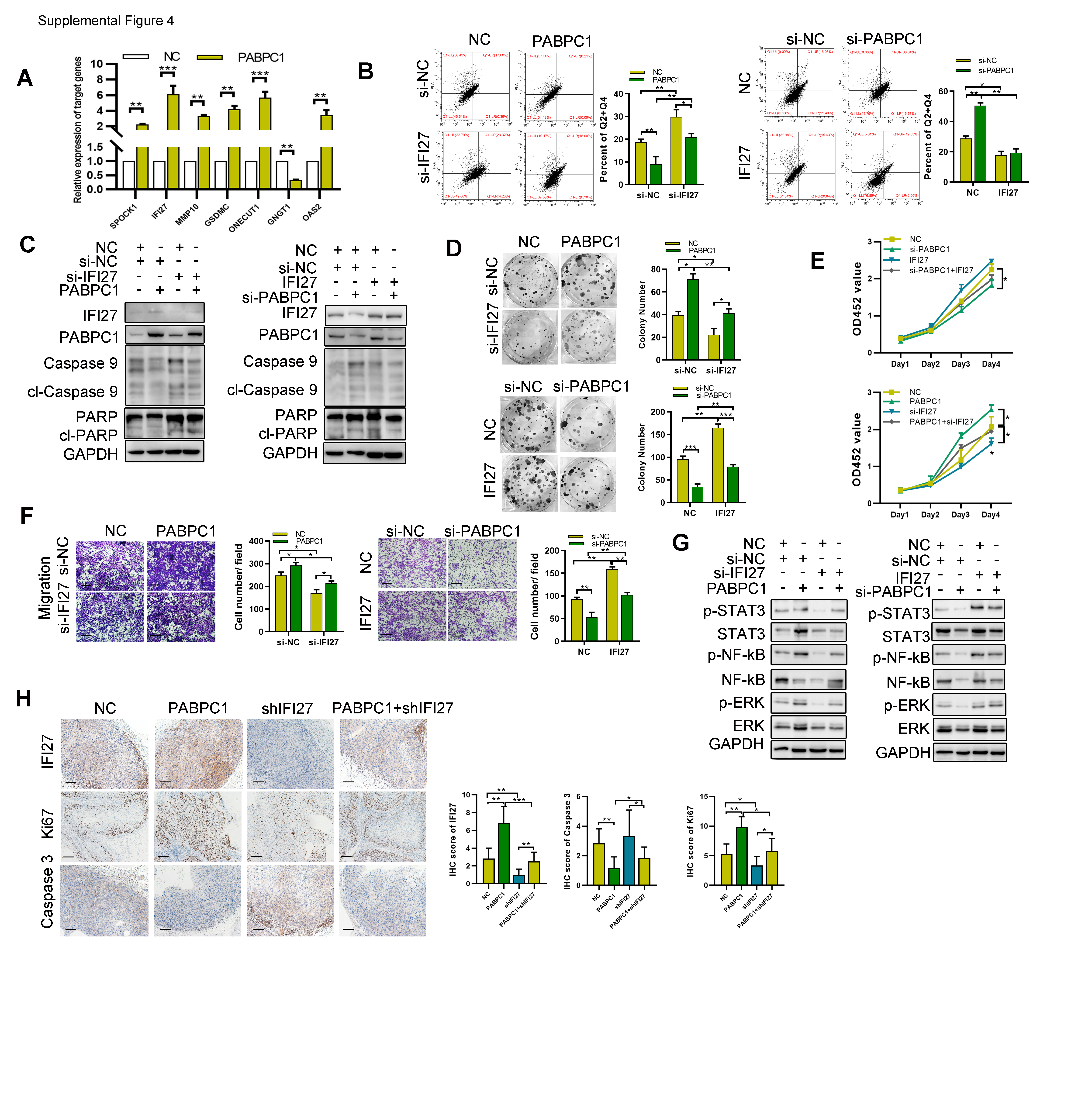

Supplement: Supplementary file 1 — Additional file 1. [file 13046_2022_2339_MOESM1_ESM.zip › supplemental figure 4-rescue.tif]

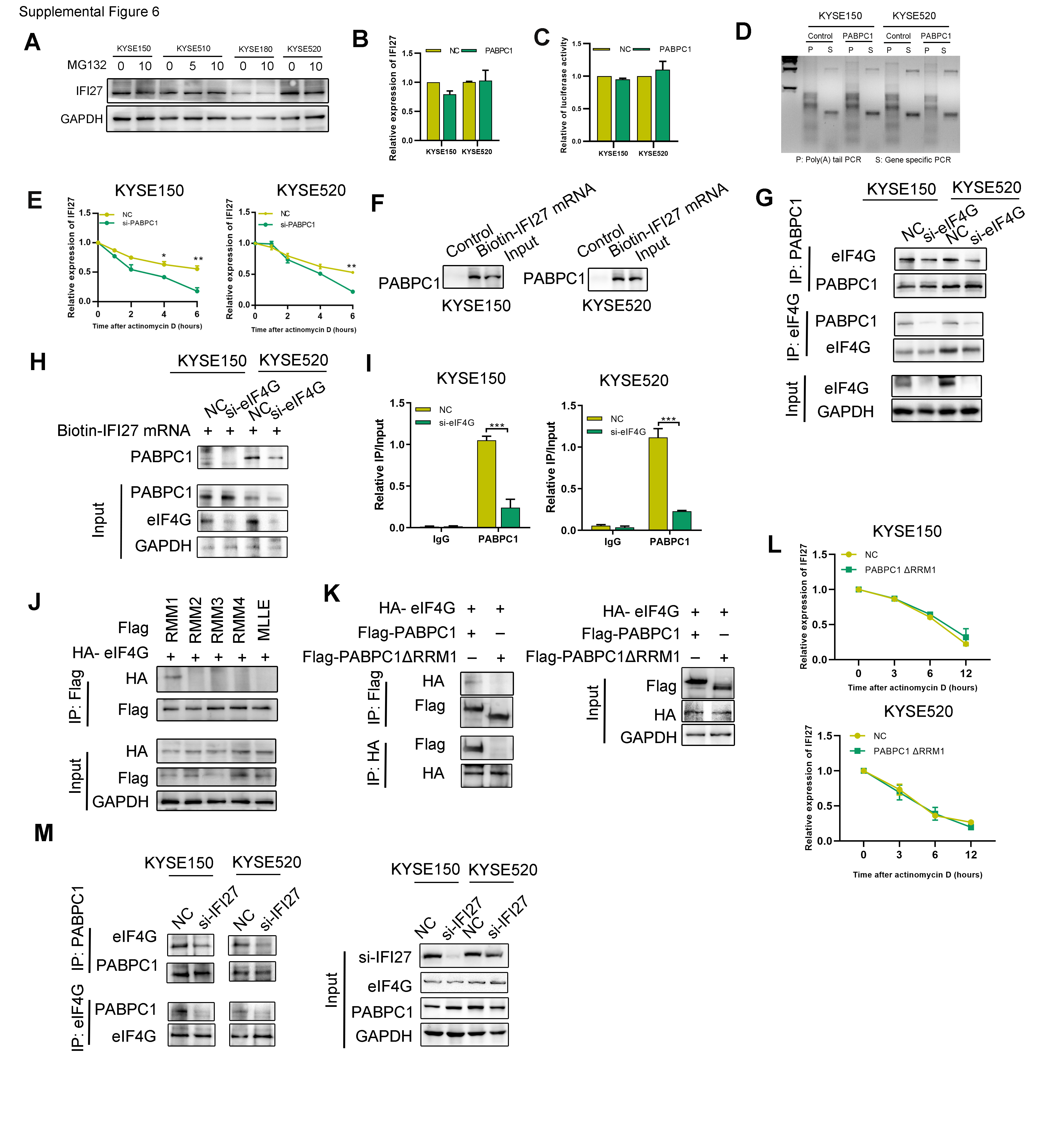

Supplement: Supplementary file 1 — Additional file 1. [file 13046_2022_2339_MOESM1_ESM.zip › SUPPLEMENTAL FIGURE 6-IFI27FUNCTION.tif]

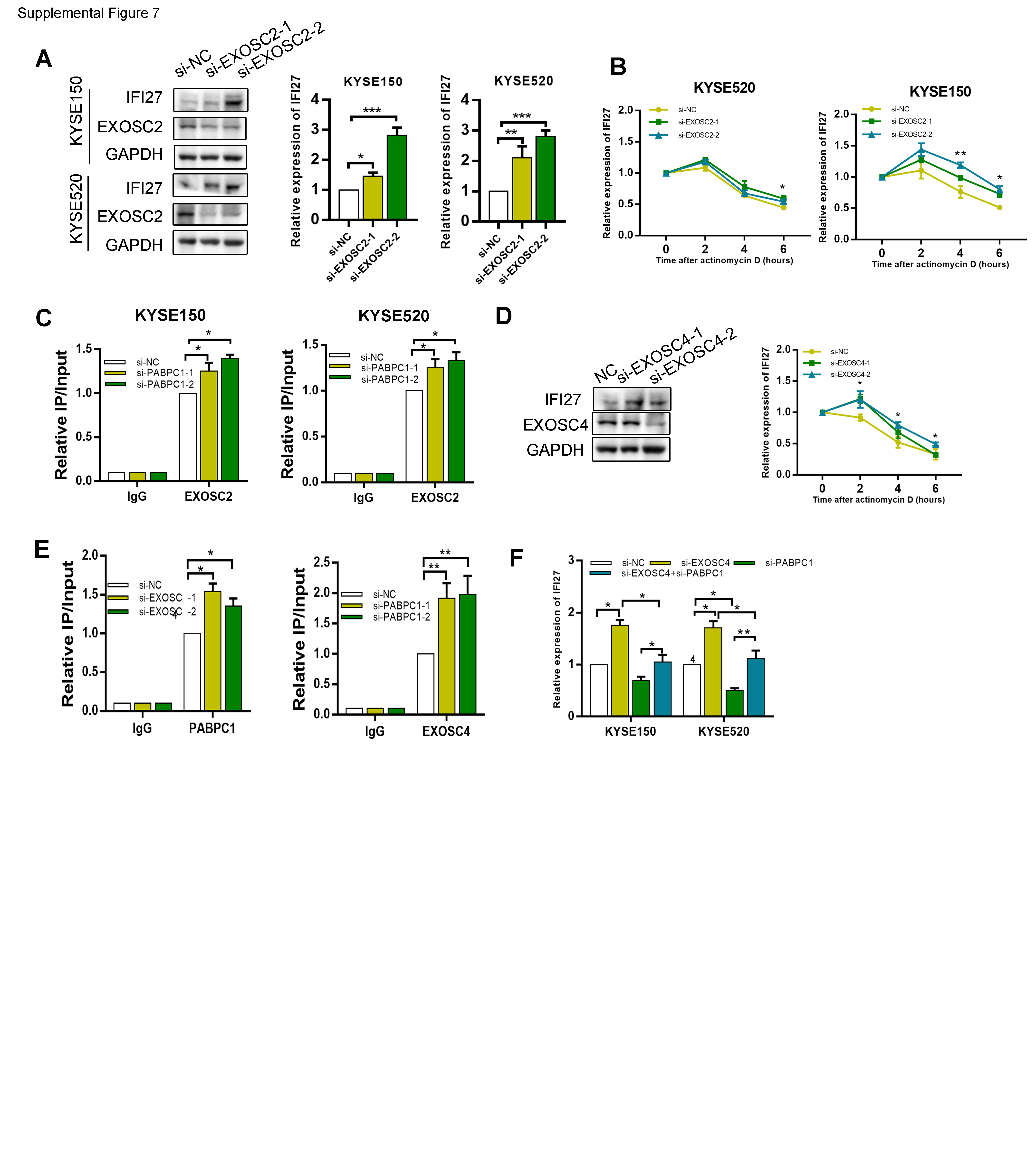

Supplement: Supplementary file 1 — Additional file 1. [file 13046_2022_2339_MOESM1_ESM.zip › Supplemental Figure 7-RNA exosome.tif]
